# Comprehensive genetic diversity revealed in the pre-breeding RILs (*O. sativa* × *O. rufipogon*) with enhanced yield and pigmented grain quality

**DOI:** 10.3389/fgene.2025.1659937

**Published:** 2025-10-01

**Authors:** Subhas Chandra Roy, Pankaj Shil

**Affiliations:** Plant Genetics and Molecular Breeding Laboratory, Department of Botany, Rice Germplasm Conservation and Breeding Centre, University of North Bengal, Siliguri, India

**Keywords:** Rice pre-breeding, *Oryza rufipogon*, black rice RIL development, widening genetic base, anthocyanin pigment

## Abstract

Developing high-yielding rice varieties (*Oryza sativa* L.) is critical to ensure global food security. The narrow genetic base in the released rice varieties has plateaued the improvement. Considering the potentials of wild rice (*Oryza rufipogon*), two distinct recombinant inbred line (RIL) populations were developed through interspecific hybridization (BWF: Badshabhog × *O. rufipogon* and CWF: Chenga × *O. rufipogon*) to increase the genetic base *via* alien introgression of hidden genes. Genetic diversity was assessed through the following: genetic variability parameters, broad-sense heritability, Mahalanobis D^2^ test, and principal component analysis (PCA) using 15 agro-morphological characteristics that indicated enhanced genetic variation. The first four principal components (PCs) together accounted for 73.74% of the variability in BWF, and the first six PCs showed 71.90% cumulative variability in CWF (eigen value >1). The broad-sense heritability ranged from 74.42% to 99.87% for all traits in both the RILs. Single plant yield was positively correlated with grain per panicle, 1,000 grain weight, grain length, and panicle weight. The cluster analysis showed that the grain per panicle, grain weight, kernel breadth, and plant height were the key yield-contributing traits. The detection of petunidin 3-O-glucoside through HR-LCMS-QTOF indicated that anthocyanin was synthesized in the black-grain RILs, signifying nutritional improvement. Hence, underutilized wild rice contributed immensely to enhancing the genetic base of the RILs, with unusual genetic diversity associated with yield improvement and grain pigmentation. Pre-breeding materials are the cornerstone of future rice improvement programs, and our materials can be efficiently utilized to develop resilient, productive, and nutritious pigmented rice varieties.

## Introduction

Rice (*Oryza sativa* L.) is the single most important crop of the world, as half of the world’s population (>3.5 billion) eats rice every day, and it contributes to global food and nutritional security (Gross and Zhao, 2014; Rao et al., 2020; Alam et al., 2024; Huang et al., 2024). The majority of the production and consumption is dominated by Asian countries, with approximately 90% of the total production and consumption occurring in Asia (FAO, 2024). Rice contributes about 20% of the world’s dietary energy supply, whereas wheat and maize contribute 19% and 5%, respectively (GRiSP, 2013; Bin Rahman and Zhang, 2022; FAO, 2024). In some Asian countries, rice provides over 30%–80% of calorie supply, and it is considered a major source of staple food for Asia (Laghari et al., 2025). The need to produce rice will double by 2050 to feed the more than 9 billion people in this world, even while simultaneous factors, such as diminishing acreage, deteriorating soil health, and environmental stresses induced by global climate change (Gaikwad et al., 2021; Xu et al., 2021; Malik et al., 2022; Seck et al., 2023), will intensify. Undoubtedly, plant breeders have witnessed a substantial increase in yield over the years during the green revolution era, but a major bottleneck has been the significant reduction in genetic diversity due to limited utilization of genetic resources. Consequently, the narrow genetic base of improved varieties led to yield plateaus (Tanksley and McCouch, 1997; Tian et al., 2006; Sanchez et al., 2013; Seck et al., 2023). Moreover, a genetic bottleneck that occurred during the domestication of cultivated rice from its immediate ancestral progenitor, the wild rice *Oryza rufipogon*, has played a major role in reducing the allelic diversity, at least by 50%–60% in cultivated rice compared to that of wild rice *O. rufipogon*, leading to loss of genetic variability along with yield potentiality (Xiao et al., 1996; Tanksley and McCouch, 1997; Brar and Khush, 2006). Wild rice *O. rufipogon* has been considered a reservoir of many untapped gene/quantitative trait loci (QTLs) for important agronomic traits such as yield, quality, nutritional characteristics, and resistance to biotic and abiotic stresses (drought, salinity, submergence, and aluminum toxicity). It can be utilized in the pre-breeding program for broadening the genetic base of released varieties to break the yield plateaus (Tanksley and McCouch, 1997; Sun et al., 2001; Tester and Langridge, 2010; Sanchez et al., 2013; McCouch et al., 2007; Brar and Khush, 2018; Solis et al., 2020; Siddiq and Vemireddy, 2021; Padmavathi et al., 2024). The transfer of genes controlling desirable traits (yield and grain quality) from wild relative *O. rufipogon* to cultivated rice is an important strategy in rice breeding (Tian et al., 2006; Huang et al., 2012b; Qiao et al., 2016; Zhang B. et al., 2022). The introgression of superior alleles from diverse sources of wild rice has been shown to widen the gene pool of cultivated rice to breed cultivars with improved yield, quality, and stress tolerance (Xiao et al., 1996; Subudhi et al., 2015; Singh et al., 2020; Gaikwad et al., 2021; Malik et al., 2022; Siddiq and Vemireddy, 2021; Zhang B. et al., 2022; Cao et al., 2022). Yield-related QTLs have been transferred from *O. rufipogon* to cultivated rice for yield enhancement through interspecific hybridization (pre-breeding) (Thomson et al., 2003; McCouch et al., 2007; Imai et al., 2013; Siddiq and Vemireddy, 2021; Eizenga et al., 2024). However, breeders are facing not only the yield barrier but also the quality improvement barrier. Because white rice provides food for approximately 3.5 billion people, its nutritional quality is poor compared to that of pigmented rice (Ito and Lacerda, 2019; Mbanjo et al., 2020). The nutritional quality of rice is determined by the levels of starch, protein, lipids, minerals, vitamins, and phytochemicals (Senguttuvel et al., 2023). Pigmented rice varieties (brown, red, and black) are gaining popularity among consumers due to their nutritional health benefits (Shao et al., 2018; Mendoza-Sarmiento et al., 2023; Xie et al., 2024; Zhu et al., 2024; Idrishi et al., 2024; Sakulsingharoj et al., 2024; Gogoi et al., 2024), and market demands are expected to increase (Kushwaha, 2016; Ito and Lacerda, 2019; Bhuvaneswari et al., 2023). Pigmented rice accumulates various types of secondary metabolites, such as phytosterols, polyphenols, flavonoids, anthocyanins, proanthocyanidins, vitamins, and micronutrients, which are known to have a high nutritional value and medicinal properties (Shao et al., 2018; Mbanjo et al., 2020; Zhu et al., 2024; Idrishi et al., 2024; Thilavech et al., 2025). Purple and red rice extracts inhibited the viability of human colorectal cancer cell line SW480 at 24-h and 48-h exposures starting at doses of 0.5 mg/mL and higher. The red varieties had higher bioactivity than purple varieties, whereas non-pigmented rice displayed no effect on cell viability (Rao et al., 2019; Brotman et al., 2021). Ghasemzadeh et al. (2018) demonstrated that a level of 119.2 mg/mL–148.6 mg/mL of black rice extracts and 151 mg/mL–175 mg/mL of red rice extracts exhibited potent antiproliferative activity against MCF-7 and MDA-MB-231 breast cancer cell lines (Ghasemzadeh et al., 2018; Tiozon et al., 2023; Das et al., 2023). Reactive oxygen species (ROS) and reactive nitrogen species (RNS) are majorly produced in the body due to induced oxidative stress, leading to carcinogenesis, aging, and inflammation. Thus, anthocyanin, which is the major polyphenol pigment present in black rice, scavenges the ROS and RNS produced in cells (Ito and Lacerda, 2019). Mapoung et al. (2022) reported that the main anthocyanin components present in bran and germ of black rice, viz. cyanidin 3-glucoside and peonidin 3-glucoside, have the ability to inhibit inflammation, leading to infections caused by the spike glycoprotein of SARS-CoV-2 virus. Black rice phenolics (BRPs) are helpful in managing type 2 diabetes mellitus (T2DM) in rats. The results indicated that BRPs significantly alleviated diabetic symptoms, lowered the fasting blood glucose and hemoglobin A1c (HbA1c) levels, and enhanced glucose tolerance in T2DM rats (Xu et al., 2024a). The antidiabetic effects of pigmented rice appear to arise from a synergistic effect of anthocyanin, proanthocyanidin, vitamin E, gamma-oryzanol, and various flavonoids, and they inhibit alpha-glucosidase and alpha-amylase activity, thus delaying the absorption of carbohydrates while tested on streptozotocin-induced diabetic rats (Tantipaiboonwong et al., 2017). The nutritive value of pigmented rice is greatly influenced by genetics, genotypic variation, and environmental factors (Shirley, 1998; Sweeney et al., 2006; 2007; Furukawa et al., 2007; Gross and Zhao, 2014; Tiozon et al., 2023; Zhu et al., 2024), along with several external influences, such as soil fertility status, the degree of milling, and the method of preparation before consumption (Gogoi et al., 2024). Numerous black rice lines were developed through crossing between black rice Okunomurasaki and white rice Koshihikari and black rice Hong Xie Nuo with white Koshihikari (Maeda et al., 2014). Transgressive segregant lines were selected from the crosses between black rice Chakhao Poireiton and white rice Sahbhagi Dhan, with colored pericarp, high anthocyanin content, and increased yield compared to their parental lines (Lap et al., 2024). Looking forward, there is a remarkable opportunity for breeding programs to develop nutritionally enriched (phytonutrients), productive pigmented rice varieties. Despite having nutritional importance (rich source of phytonutrients), pigmented rice is usually low yielding, prone to lodging, susceptible to diseases, and late-maturing (Devi et al., 2020; Bhuvaneswari et al., 2020; Sedeek et al., 2023). The narrow genetic base of modern rice varieties has led to yield plateaus, making it essential to introduce genetic diversity to overcome these barriers. Pre-breeding involves crossing elite cultivars with wild relatives to incorporate novel genes and QTLs for the improvement of traits. Pre-breeding facilitates the introgression of desirable traits such as yield potential, nutritional quality, and resistance to biotic and abiotic stresses (Rao et al., 2020; Bin Rahman and Zhang, 2022; Alam et al., 2024). Therefore, the present study aimed to broaden the genetic base through interspecific hybridization between the rice cultivars Badshabhog and Chenga with wild rice *O. rufipogon* to increase yield potential and enhance grain quality.

## Materials and methods

### Plant materials for interspecific hybridization

Wild rice *O. rufipogon* Griff. of Raiganj was used as one of the parental lines (donor parent). This wild rice variety grows naturally in the shallow marshy land/ditches of the Raiganj block, Uttar Dinajpur district, West Bengal, India, at latitude 25.62 °N and longitude 88.12 °E, and elevation of 40 m (130 ft). This wild rice variety is highly shattering in nature and fully spreading in the habitat with an annual growth pattern, and it produces awned spikelets on the spreading panicles and disperses the mature seeds. Its grain and hull colors are red and black, respectively. Two well-adapted farmer’s varieties of *O. sativa* L. subspecies *indica* cultivar, Badshabhog and Chenga, were used as the parents in this interspecific hybridization. Badshabhog has white aromatic grains with a straw-colored husk, whereas Chenga has a blackish husk with nonaromatic brown grains. Both the farmer’s rice varieties (Badshabhog and Chenga) have been deposited in the National Rice Gene Bank, NBPGR-ICAR, Govt. of India, New Delhi, for conservation purpose with indigenous collection numbers (IC No-0652950 for Chenga and IC no-0652952 for Badshabhog).

### Development of RILs following the pedigree method

#### Interspecific hybridization between cultivated rice (*O. sativa*) and wild rice (*O. rufipogon*)

Two recombinant inbred line (RIL) populations were developed through interspecific hybridization between cultivated rice varieties (*O. sativa*) and wild rice (*O. rufipogon*). The RIL population developed from the cross Badshabhog × *O. rufipogon*” (named BWF) comprised 100 distinct individual lines in the F7 generation, whereas the population developed from the cross “Chenga × *O. rufipogon*” (named CWF) comprised 100 individual genotypes in the F7 generation. In the pedigree method, individual F2 generation plants were carefully selected, with their cultivated offspring, and a detailed pedigree record was maintained following the standard method (Xu et al., 2024b). The process began by crossing cultivated rice varieties (*O. sativa*) with wild rice *O. rufipogon*. Initial crosses were made between *O. sativa* cv. Badshabhog × *O. rufipogon* and *O. sativa* cv. Chenga × *O. rufipogon* for the creation of F1 progenies in 2016 according to the standard protocols, and they were collectively planted and harvested (Sleper and Poehlman, 2007; Sha, 2013; Roy, 2017).

RIL population development was carried out at the NBU, Siliguri, India, beginning with a cross between Badshabhog and Chenga as the female parent and *O. rufipogon* as the male parent. Crossability was determined by counting the number of seeds produced per cross. It was calculated as the ratio of the number of true F1 seeds developed per cross to the total number of spikelet emasculated and was expressed as the percentage.

Crossability=Total F1 seeds produced/Total number of spikelet emasculated×100.

Twelve hybrid F1 viable seeds were obtained from crossing Badshabhog × *O. rufipogon* and nine F1 hybrid seeds were obtained from crossing Chenga × *O. rufipogon*, with seed set percentages of 14.28% and 12.32%, respectively. The highest percentage of seed set among *O. sativa* × *O. rufipogon* was found in the cross BWF Badshabhog × *O. rufipogon* (14.28%) [12/84 × 100 = 14.28%], whereas the lowest crossability was exhibited by the cross CWF Chenga × *O. rufipogo*n (12.32%) [09/73 × 100 = 12.32%]. The harvested viable F1 seeds from both BWF (12 F1 seeds) and CWF (nine F1 seeds) crosses were sown to obtain the next generation, F2. Due to the hybrid sterility/hybrid breakdown criterion, a few F1 plants could not grow properly and died (two in BWF and one in CWF). The remaining 10 F1 progeny in BWF plants and eight F1 progeny in CWF plants were developed after overcoming hybrid sterility, and 174 F2 seeds were collected from each of the two crosses. F2 plants were randomly selected to proceed to F3, and 50 F2 plants were selected based on the phenotype to eliminate non-desirable characteristics, including very late- or non-flowering types, excessively tall plants, and sterile plants. A total of 243 F_3:4_ plants were selected and analyzed. F2 plants were individually grown to identify and select optimal lines. All F2 seeds were highly shattering and collected in a nylon net by bagging the panicles to harvest the seeds. This selection process was extended to the F3 generation, where individual plant progeny was row-planted, and optimal plants were selected. Progeny populations from the F2 generation (in 2017) were allowed to self-fertilize to develop RILs. Shatteredness was reduced from the F3 and F4 generations, and from the F5 generations, shattering was stopped in most of the breeding lines and maintained. This selection procedure was continued until the production of the F6 generation. From the F6 populations (2021 kharif crop), we selected 100 phenotypically distinct breeding lines based on 15 yield-related traits from each of the populations (BWF and CWF). To establish the RIL population following the pedigree method, 100 superior progeny plants were collectively harvested from the F7 generation, at which point they achieved more uniformity in trait expression.

#### Experimental design

Yield trials were conducted during two consecutive kharif seasons of 2022 and 2023. The parents and both RIL populations were grown at the experimental rice field of North Bengal University. Genotypes were plotted in the field in a randomized complete block design (RCBD) with three replications for two seasons (F7 in 2022 and F8 in 2023 kharif crop) to evaluate the yield performance. Local black rice cultivar Chakhao was used as the control variety. The plot size was 6 m^2^ (2.0 m × 3.0 m). The 30-day-old seedlings were transplanted with a spacing of 20 cm × 20 cm and one seedling per hill. The fertilizer application and intercultural agronomic practices were carried out as per the recommended standard.

### Trait measurement

#### Phenotypic evaluation

The phenotypic evaluation of both the RIL populations containing 100 genotypes (BWF and CWF) was performed under natural conditions at the experimental field of North Bengal University in two kharif seasons of 2022 and 2023. Phenotypic data regarding 15 yield and yield-related traits, including plant height (PH), flag leaf length (FFL), flag leaf width (FLW), panicle length (PnL), panicle weight (PnWt), grain per panicle (GrPn), grain length (GL), grain breadth (GB), kernel length (KL), kernel breadth (KB), 1,000 grain weight (GrWt), tiller number (Till), heading date (HD), maturity time in days (MT), and single plant yield (PY), were recorded from five randomly selected representative plants in each plot of each replication using the DUS guideline (PPV&FR Act, 2001, Govt. of India). Data were recorded from the middle rows to avoid border effects, and the mean values of the 15 traits were used for further analysis. Other agro-morphological and grain quality parameters such as awn length (AwnL), aroma (Aroma), ASV, GT, and GC; pericarp pigmentation color (PC); seed shattering habit (Sh); and seed coat phenol test were recorded and analyzed.

### Statistical data analysis and genetic diversity studies

The mean pooled data obtained from two kharif seasons (2022 and 2023) were used for biometrical analysis. The genotypic and phenotypic variation, broad-sense heritability, genetic advance, and genotypic and phenotypic correlation coefficients were estimated. Genetic diversity analysis was performed following D^2^ statistics proposed by Mahalanobis (1936). The RIL genotypes were classified into several clusters by Tocher’s method using Mahalanobis D^2^ distance statistics (Rao, 1952). The broad-sense heritability (H%) and other genetic variability parameters were calculated using the standard methods (Johnson et al., 1955; Allard, 1960). Phenotypic coefficients of correlation were calculated based on Burton and de Vane’s formula (1953). The multivariate PCA was utilized to estimate the relative contribution of various traits to the total variability based on the original concept of Pearson (Hotelling, 1933). Statistical analyses were carried out using various software applications, such as SPSSv-22, XLSTAT, PAST4.03, Origin 2024, and R4.4.1.

The following formulas were used to calculate the genetic variability parameters:

### Heritability (broad-sense) measurement (H%):

Broad-sense heritability of the breeding lines was estimated using the formula by Allard (1960).

$$\text{Broad sense heritability }\left(H\right)=\frac{\sigma^{2}g}{\sigma^{2}p}\times 100.$$

where (H) = broad-sense heritability; σ^2^p = phenotypic variance; σ^2^g = genotypic variance. Genotypic variance (σ^2^g) = (MS_2_ –MS_3_)/b; error variance (σ^2^e) = MS_3_; MS_2_ = mean square of populations; MS_3_ = mean square of error; b = number of blocks.

$$\text{Phenotypic variance }\left(\sigma_{p}^{2}\right)=\sigma_{g}^{2}+\sigma_{e}^{2}.$$

$$\text{Genotypic coefficient of variation }\left(\text{GCV}\right)=\sqrt{\sigma_{g}^{2}/X^{-}}\times 100.$$

$$\text{Phenotypic coefficient of variation }\left(\text{PCV}\right)=\sqrt{\sigma_{p}^{2}/X^{-}}\times 100.$$

X^¯^ = the mean of the trait.

Different variance components such as the phenotypic coefficients of variation (PCV) and genotypic coefficient of variation (GCV) were estimated according to the method of Burton and de Vane (1953). Environmental variance was calculated by the formula suggested by Burton and de Vane (1953). The estimation of genetic advance (GA) and the genetic advance as percentage of the mean (GAM) were calculated according to the method described by Johnson et al. (1955).

$$\text{Genetic Advance }\left(\text{GA}\right)=\frac{k\times \sigma^{2}p\times \sigma^{2}g}{\sigma^{2}p}.$$

$$\text{Genetic Advance as Percentage of Mean }\left(\text{GAM}\right)=\frac{\text{GA}}{X}\times 100.$$

where GA = genetic advance; K = standardized selection differential at 5% selection intensity (k = 2.063); σ^2^p = phenotypic variance; σ^2^g = genotypic variance; GAM = genetic advance as percentage of the mean; × = grand mean of a character.

### Physicochemical properties and sensory-based aroma test

The alkali spreading value (ASV) (on a scale of 1–7) was measured according to the standard method (Little et al., 1958). A low ASV corresponds to a high gelatinization temperature (GT), and conversely, a high ASV indicates a low GT (Little et al., 1958). Sensory-based aroma (on a scale of 0–3) was evaluated using the standard procedure (Sood and Siddiq, 1978). The gel consistency (GC) was measured as per the standard protocol (Little et al., 1958).

### Phenol reaction of seed coat

The phenol reaction of the seed coat was tested according to the protocol (Kumar et al., 2021). Freshly harvested grains were collected. Fifteen healthy grains of each cultivar and breeding lines were soaked in 1.5% aqueous phenol solution for 24 h. After that, the solutions were drained, and the grains were air-dried. The hull color was then recorded unstained and stained as compared to the control treatment, in which the grains were treated with distilled water.

### Metabolomics analysis of grain quality through the HR-LCMS-QTOF method

Anthocyanin pigments were qualitatively identified from the grains of black rice lines (BW23 and CW16) according to the standard methods (Bhuvaneswari et al., 2020). In brief, the dried, pigmented black rice grain samples (1g) were ground with 5 mL of 70% aqueous methanol at room temperature. After centrifugation at 10,000 × *g* for 10 min, the extracts were filtered (0.22 μm) before HR-LCMS-QTOF analysis at the SAIF, IIT Bombay, India. The instrument used was HRLCMS QTOF (Agilent Technologies, United States), the data acquisition software was Agilent MassHunter, and the data processing software was Agilent MassHunter Qualitative Analysis B.06; the column was an ZORBAX Eclipse Plus-C18 150 × 2.1 MM, 5 microns (Agilent). The following solvents were used: solvent A: 0.1% formic acid in Milli-Q water and solvent B: acetonitrile. The instrument scanned over the mass (m)/charge (z) range of 100–1,100 in both the positive and negative ion modes.

### Identification of amino acids in black lines using HR-LC/MS-QTOF

Total amino acid (TAA) identification was performed using standard protocols (Liyanaarachchi et al., 2020; Tyagi et al., 2022). Briefly, 100 mg of black rice flour (BW23 and CW16) were hydrolyzed in 10 mL of 6N HCI at 110 °C for 24 h. Approximately 20 µL of the solution was taken from the hydrolyzed samples and evaporated by speed vac. Then, it was reconstituted by adding 50 µL of 0.1 N HCl. From this extract, 1 µL of the sample was loaded into the LCMS system for amino acid profiling, along with standard amino acids. Amino acid identification and quantitative analysis were performed with an HR-LCMS-QTOF mass spectrometer (Agilent Technologies, United States; SAIF, IIT Bombay, India) with the following parameters: dual ion source AJS ESI, HiP sampler, binary pump, and diode-array detection (DAD) with gradient elution in a Q-TOF column comp (Poroshell HPH-C18, 2.7 µ, 4.6 × 100 mm).

## Results

### Phenotyping for yield and yield-related traits in RIL populations

**FIGURE 1:**
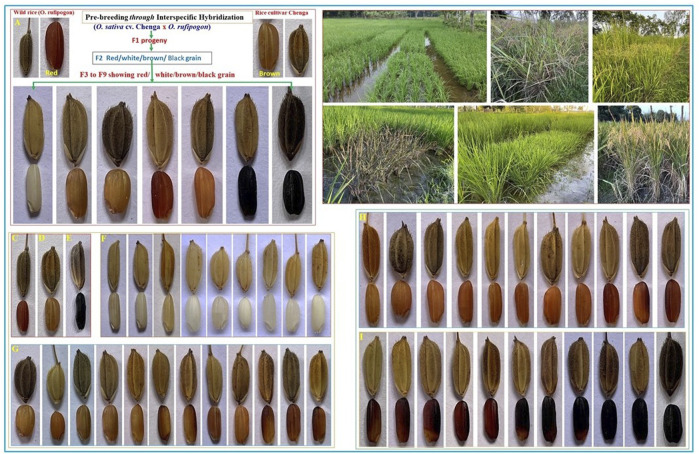
Scheme for recombinant inbred line (RIL) development in the pre-breeding program and the field trial of both the RIL (CWF and BWF) populations with grain color variations. Demonstration of the development of the CWF RIL population: interspecific hybridization was made in 2016 between *Oryza sativa* ssp. indica cv. Chenga and Asian common wild rice *Oryza rufipogon* Griff., and then the F1 generation was allowed to self-fertilize up to the seventh generation for the generation of the RIL population. RIL genotypes showing different grain colors including black pericarp in the F2 generation and inherited till the F6 (2021), F7 (2022), and F8 (2023). Field trial and experimental design: progeny lines were plotted in the field in a randomized complete block design (RCBD) with three replications for two seasons (F7 in 2022 and F8 in 2023 kharif crop) to evaluate the yield performance; the purple leaf plot is also shown. Wild rice *Oryza rufipogon*, with red grain and black husk with long awns. Rice cultivar Chenga, with brown grain and blackish husk, Chakhao control black rice, with black grain and black husk. White grain series with the size and husk color of pre-breeding F8 lines. Brown grain series with the size and husk color of pre-breeding F8 lines. Red grain series with the size and husk color of pre-breeding F8 lines. Black grain series with the size and husk color of pre-breeding F8 lines.

We have developed two RIL populations (100 genotypes in each population) through interspecific hybridization, namely, BWF (*O. sativa* cv. Badshabhog × *O. rufipogon*) and CWF (*O. sativa Cv. Chenga* × *O. rufipogon*), to enhance the genetic base of the rice cultivars. In our cross, we have used only AA genome-containing genotypes within the primary gene pool of Oryza species (*O. sativa*, 2n = 24, AA genome and *O. rufipogon*, 2n = 24, AA genome), which is why the progeny lines (F1) showed recombination stability; however, hybrid sterility/hybrid breakdown was sometimes observed in the F1 generation. Due to this hybrid sterility/hybrid breakdown criterion, many F1 plants could not grow properly and died. In this study, the analysis of variance (ANOVA) revealed significant (p < 0.001) differences among the 100 RILs for all 15 measured yield-related agro-morphological traits (Table 1). These findings indicate the presence of ample genetic variations among the genotypes in the RIL populations. The 15 yield-related agro-morphological traits were evaluated for two consecutive kharif seasons (2022 and 2023), and some of the RIL genotypes exhibited superior performance in terms of PY and with pigmented grain quality (Tables 2, 3). An unexpected range of phenotypic variation was recorded among the RILs of BWF and CWF (Figure 1; Tables 2, 3). The shortest PH of only 60 cm was recorded in the BW98 line with a small FFL of 15.17 cm and a width of only 5.07 mm with small shattered seeds (Table 3). In contrast, the tallest PH (204 cm) was observed in line BW97 with shattered seeds. The grain pericarp color in both the RILs (BWF and CWF) varied from white, brown, red, and greenish to black, with distinctive grain quality parameters viz.*,* ASV, GT, GC, and aroma (Figure 1; Supplementary Tables S1, S2).

**TABLE 1:** Mean squares of the analysis of variance (ANOVA) for 15 yield-related agro-morphological characteristics for both the RIL populations (BWF and CWF).

BWF RILs consisting of 100 genotypes (Badshabhog × *O. rufipogon*)																
Source of variation	df	Mean sum of square (MS)														
		PH(cm)	FLL (cm)	FLW (mm)	PnL (cm)	PnWt(g)	GrPn	GrWt (g)	GL (mm)	GB (mm)	KL (mm)	KB (mm)	HD (days)	MT (days)	Till	PY (g)
Genotype	102	2,341.04	216.52	35.81	94.18	8.84	41,039.61	252.25	13.01	0.84	7.88	0.28	529.21	480.7	38.55	1,194.38
Error	927	7.17	2.04	0.24	1.51	0.12	195.22	0.16	0.06	0.03	0.05	0.02	0	0	0.6	1.99
Replication	6	22.468*	2.154*	0.231*	0.884**	0.091*	50.874*	0.921*	0.011*	0.004 ns	0.005 ns	0.001 ns	0.874*	1.742*	0.602**	2.084*
Genotypes	102	152.347***	28.941***	1.846**	4.833***	0.842*	629.512***	8.412***	0.284*	0.029 ns	0.034 ns	0.009 ns	12.365***	15.124***	4.958***	18.693***
Location	2	1,875.642***	420.512***	15.217***	65.121***	3.916***	5,112.874***	75.362***	0.624***	0.112***	0.198***	0.024***	46.214***	84.693***	27.658***	144.751***
Genotype × environment	204	45.231***	6.942***	0.524***	1.923***	0.214***	105.421***	1.945***	0.017*	0.006*	0.007 ns	0.001 ns	1.748***	3.471***	1.229***	4.285***
Error	612	10.452	1.028	0.115	0.436	0.047	25.412	0.481	0.007	0.002	0.003	0.000	0.432	0.869	0.301	1.041
CV%		10.93	15.41	2.68	11.96	28.22	35.54	23.93	13.73	10.78	14.3	9.87	7.43	0.03	16.44	38.31
LSD(0.05)		2.34	1.25	0.42	1.07	0.3	12.26	0.35	0.21	0.14	0.19	0.1	0.03	0.01	0.68	1.23
SS between group		238,786.56	22,085.13	3,652.91	9,607.06	901.72	4,186,040.5	25,729.22	1,326.99	85.39	804.62	28.71	53,979.7	49,032.37	3,932.91	121,827.7
SS within group		6,644.75	1,887.77	221.87	1,403.11	111.9	180,965.12	149.79	53.61	24.66	48.13	14.29	1.09	0.09	559.88	1,848.4
Significant		***	***	***	***	***	***	***	***	**	*	*	***	***	***	***

CWF RILs consisting of 100 genotypes (Chenga × *O. rufipogon*)																
Source of variation	df	Mean sum of square (MS)														
		PH (cm)	FLL (cm)	FLW (mm)	PnL (cm)	PnWt (g)	GrPn	GrWt (g)	GL (mm)	GB (mm)	KL (mm)	KB (mm)	HD (days)	MT (days)	Till	PY (g)
Genotype	102	1,529.69	212.44	24.47	66.22	7.95	6,907.48	86.08	2.76	0.44	2.52	0.35	262.53	148.06	48.27	495.9
Error	927	2.93	1.86	0.19	0.93	0.09	39.25	0.17	0.07	0.06	0.04	0.01	1.27	1.91	2.32	2.49
Replication	6	21.975*	2.138*	0.229*	0.876**	0.089*	49.762*	0.912*	0.01*	0.003 ns	0.005 ns	0.001 ns	0.862*	1.721*	0.595**	2.061*
Genotypes	102	148.562***	27.914***	1.792**	4.624***	0.816*	612.385***	8.221***	0.276*	0.028 ns	0.033 ns	0.009 ns	11.984***	14.872***	4.868***	18.111***
Location	2	1,820.451***	408.227***	14.798***	63.434***	3.827***	4,962.742***	74.001***	0.612***	0.109***	0.196***	0.023***	45.336***	82.887***	27.181***	142.305***
Genotype × environment	204	44.521***	6.835***	0.519***	1.899***	0.21***	103.626***	1.918***	0.017*	0.006*	0.007 ns	0.001 ns	1.722***	3.434***	1.215***	4.239***
Error	612	10.313	1.019	0.114	0.432	0.046	24.971	0.478	0.007	0.002	0.003	0.000	0.428	0.862	0.298	1.032
CV%		8.72	14.95	10.73	9.99	30.98	26.67	13.01	5.96	6.82	7.71	8.64	4.94	2.8	18.77	31.44
LSD (0.05)		1.5	1.19	0.38	0.84	0.07	5.49	0.36	0.04	0.03	0.03	0.02	0.98	1.21	1.33	1.38
SS between group		156,028.51	21,668.98	2,496.83	6,754.51	811.59	704,563.07	8,781.02	282.05	45.72	257.03	36.45	26,778.5	15,102.78	4,924.39	50,582.27
SS within group		2,718.06	1,728.33	178.08	863.37	7.03	36,391.09	164.05	2.36	1.28	1.63	0.9472	1,178.65	1,771.02	2,155.76	2,313.52
Significant		***	***	***	**	***	***	***	***	**	*	*	***	***	***	***

*, **, and *** significant at 5%, 1%, and 0.1% levels of probability, respectively.

Abbreviations: ns, nonsignificant; PH, plant height (PH); FFL, flag leaf length; FLB, flag leaf breadth; PnL, panicle length; PnWt, panicle weight; GrPn, grain per panicle; GL, grain length; GB, grain breadth; KL, kernel length; KB, kernel breadth; GrWt, 1,000 grain weight; Till, tiller number; HD, heading date; MT, maturity time in days; PY, single plant yield.

**TABLE 2:** Mean performance of yield and yield related 15 agromorphological traits in 100 BWF RIL lines.

Rice Breeding lines	PH (cm)	FLL (cm)	FLW (mm)	PnL (cm)	PnWt (g)	GrPn	GrWt (g)	GL (mm)	GB (mm)	KL (mm)	KB (mm)	HD (days)	MT (days)	Till	PY (g)
Chakhao (Control Black rice)	156.55	45.51	14.37	25.67	2.89	86.82	23.28	8.76	3.12	6.68	2.09	112	153	8.57	14.95
Badshabhog (Parent 1)	148.29	34.27	11.63	28.11	2.28	263.41	10.67	6.17	1.98	4.53	1.34	109	148	12.38	22.65
*O. rufipogon (Parent 2)*	163.29	20.28	7.69	18.89	1.08	26.77	17.38	8.48	2.08	6.71	1.42	127	157	20.85	10.66
BW1	149.9	36.1	17.73	24.64	3.26	155.45	23.35	8.75	3.02	6.61	2.04	110	150	12.22	26.11
BW2	157.23	30.42	13.85	25.54	4.07	199.29	24.38	8.9	3.16	6.29	2.01	110	148	13.97	42.59
BW3	144.6	35.92	16.63	28.76	3.41	208.13	22.61	9.06	3.24	7.05	2.24	107	148.66	9.77	39.3
BW4	143.35	29.94	15.49	29.54	4.32	234.07	22.73	9.37	3.08	6.8	1.98	101	142	12.97	37.65
BW5	137.25	32.42	16.97	29.61	4.35	231.72	22.7	8.48	3.38	6.39	2.03	100	140	12.9	43.78
BW6	127.76	27.62	14.88	27.09	4,12	200.4	27.39	10.02	3.16	7.13	2.23	100	140	11.89	33.48
BW7	133.34	27.4	14.49	26.76	4.51	189.85	25.28	10.09	3.1	6.59	2.04	100	140	13.48	42.37
BW8	149.98	32.8	15.95	26.68	4.14	226.27	22.8	8.48	3.23	6.07	2.02	95	135	13.25	35.22
BW9	134.97	34.7	15.91	26.73	4.52	159.98	22.64	8.02	3.19	6.25	2.13	95	135	14.25	21.33
BW10	137.66	27.13	15.43	28.16	4.08	162.15	23.62	8.65	3.24	5.76	2.08	95	136.58	13.55	23.19
BW11	135.4	32.39	18.75	26.79	2.75	154.38	22.65	8.6	3.3	6.74	2.04	94.95	135.06	13.15	24.07
BW12	144.63	34.76	16.03	28.62	4.46	231.75	23.59	8.7	3.22	6.19	2.04	89.66	134.77	12.03	43.96
BW13	134.77	28.31	14.37	26.22	2.91	124.7	28.76	9.21	3.33	7.01	2.09	94.86	134.89	13.25	26.15
BW14	149.39	35.99	16.78	27	3.3	121.43	25.74	8.61	3.36	6.9	2.05	100	142.59	13.15	25.07
BW15	160.4	32.75	14.23	31.17	4.88	245.49	23.24	9.09	3.17	6.89	1.99	108	149.68	14.63	31.56
BW16	141.92	28.84	14.22	26.19	2.5	227.01	24.14	8.28	3.23	6.38	2.1	98.68	132.58	13.27	33.48
BW17	142.98	36.76	15.93	31.29	4.09	187.62	23.32	7.85	3.37	6.74	2.12	90	132.49	13.45	34.44
BW18	152.9	34.11	15.62	27.49	4.51	201.33	24.2	8.52	3.27	6.26	2.49	98.47	139.85	13.89	40.37
BW19	132.16	30.89	14.05	25.9	3.21	206.77	22.28	8.09	3.31	7.21	2.23	100	140	13.1	24.07
BW20	126.38	26.85	10.91	27.55	3.89	171.51	22.96	8.62	3.38	6.08	1.94	90	128	13.7	39.13
BW21	152.12	34.47	15.91	27.21	3.07	220.03	23.99	9.2	3.27	6.83	1.96	93.69	133.58	13.52	31.44
BW22	147.75	32.37	15.7	33.46	3.44	246.18	24.55	8.94	3.21	6.6	2.04	90	137.83	11.22	32.3
BW23	132.68	26.17	11.85	30.32	5.55	387.14	24.55	10.13	3.19	7.59	2.13	95	130	13.51	61.89
BW24	142.15	32.8	13.57	30.72	4.33	287.97	22.93	9.16	3.29	7.08	2.16	100	140	14.1	56.59
BW25	134.82	34.32	15.7	29.97	4.43	298.58	23.51	9.37	3.21	6.93	2.03	100	140	13.51	51.93
BW26	135.98	26.76	15.28	27.67	3.94	190.49	27.2	8.66	3.06	6.71	2.11	90	130	14.79	34.19
BW27	141.39	29.15	12.37	28.73	3.94	108.84	27.75	9.57	3.11	7.16	2.1	89.75	133.86	15.41	28.04
BW28	133.49	31.64	13.95	28.15	4.57	150.08	22.6	9.05	2.82	5.93	2.18	100	140	9.33	26.56
BW29	141.55	35.27	15.17	30.36	4.41	132.8	23.52	8.99	3.08	6.65	2.05	95.11	134.83	12.01	20.44
BW30	145.08	30.68	13.86	27.63	4.03	177.78	27.38	8.86	3.29	6.87	2.25	105	143.75	13.16	34.48
BW31	142.66	36.1	16.62	26.36	4.07	250.37	23	9	3.04	6.52	2.29	94.69	144.83	13.33	35.98
BW32	138.78	28.58	15.41	27.8	2.98	242.2	22.69	8.82	2.95	6.47	2.02	100	142.46	14	31.19
BW33	131.5	31.22	14.9	27.41	4.07	230.63	25.71	10	3.04	7.11	2.05	90	130	13.21	32.48
BW34	128.64	27.67	11.94	27.5	4.39	238.59	25.46	9.12	2.96	6.58	2.14	90	125	14.15	32.85
BW35	150.81	33.7	15.78	27.53	3.22	147.76	26.9	8.65	3.23	6.59	2.19	90	144.76	14.72	28.56
BW36	147.75	33.02	15.67	30.92	3.76	162.89	25.59	9.04	3.03	6.97	2.06	94.35	140.22	10.29	23.48
BW37	133.43	32.11	14.71	24.7	3.06	161.52	23.93	8.36	3.18	6.67	2.18	95	135	10.53	24.59
BW38	138.09	35.32	14.46	29.45	3.24	176.16	23.19	8.06	3.36	5.21	2.09	90	139.85	13.07	28.44
BW39	152.14	33.97	14.75	22.93	3.77	219.54	27.24	9.21	2.99	6.35	2.1	93.66	145.17	10.93	42.70
BW40	133.9	28.66	14.21	25.44	2.95	128.04	23.03	9.28	2.91	6.15	1.91	95	135	14.37	18.37
BW41	131.66	26.76	12.44	27.63	3.38	97	24.75	10.03	2.98	7.56	2.16	90	135	13.29	23.67
BW42	138.96	31.75	12.95	29.64	4.19	181.3	25.31	9.05	2.92	6.88	2.03	90	136.49	14.09	24.30
BW43	137.52	28.71	15.08	25.4	3.66	201.91	23.49	8.61	2.89	6.23	2.14	89.67	138.49	14.19	34.93
BW44	140.74	33.79	15.75	29.49	3.68	166.09	25.75	10.09	3.15	7.7	2.13	90	135	12.99	31.70
BW45	161.74	32.53	15.07	26.18	1.86	224.34	21.73	9.04	3.12	6.85	2.48	94.61	139.8	14.97	30.44
BW46	135.82	32.27	14.31	28.29	4.73	157.98	20.98	8.94	2.97	7.09	2.14	90	128.85	13.43	26.74
BW47	133.04	28.03	15.12	26.33	4.04	152.62	23.93	9.49	3.15	6.97	2.13	100	135	14.09	26.00
BW48	155.69	36.25	14.82	25.65	3.56	95.17	29.72	9.68	3.15	7.1	2.17	100	135	12.93	25.89
BW49	136.97	24.04	15.58	29.16	3.75	148.74	25.33	9.32	2.53	6.83	2.15	90	125	12.93	32.89
BW50	134.07	32.02	14.1	28.02	4.74	233.89	25.25	8.43	3.1	6.08	2.04	90	125	13.59	42.74
BW51	140.71	26.9	14.92	31.97	4.66	147.87	23.45	8.94	3.02	6.47	1.96	95	130	13.81	25.15
BW52	147.78	27.49	15.19	30.6	4.87	216.13	24.86	9.9	3.03	6.81	2.05	90	125	13.55	44.07
BW53	170.21	30.79	12.36	31.05	4.06	170.48	23.09	8.84	3.04	6.94	1.97	110	150	14.33	27.59
BW54	179.45	43.52	14.6	30.39	4.32	158.25	24.14	9.13	3.09	6.88	2.11	110	148	14.93	27.67
BW55	179.02	47.06	14.96	26.9	4.57	171.91	25.42	9.08	3.15	6.95	2.19	115	155	14.67	29.22
BW56	152.13	36.73	15.34	26.04	4.22	125.57	10.43	9.38	2.85	6.95	2.06	111	154.65	14.3	19
BW57	130.91	25.21	15.04	25.9	3.61	159.59	24.13	8.22	2.98	6.14	2.07	100	140	13.93	26.85
BW58	121.44	27.01	12.8	24.91	2.37	225.02	10.07	6.36	3.03	4.67	2.19	105	140	8.82	17.07
BW59	121.63	27.65	12.19	24.54	1.99	157.83	11.8	6.85	2.54	4.9	1.97	105	140	10.63	16.30
BW60	137.97	34.78	13.22	25.9	2.6	185.63	10.51	5.7	2.4	4.01	2.04	100.01	140.34	13.63	18.67
BW61	138.79	33.96	15.3	24.71	2.51	122.89	12.51	6.02	2.46	5.16	2.17	90	138.85	11.15	15.49
BW62	140.81	28.34	14.91	22.63	1.64	91.38	16.07	6.28	2.59	4.6	2.16	89.57	137.94	9.44	13.65
BW63	150.12	34.95	15.64	25.67	2.01	82.48	11.37	6.52	2.51	5.52	2.11	100	143.67	8.74	12.19
BW64	129.65	25.93	12.27	22.33	1.94	128.5	11.55	5.84	2.49	5.3	2.02	90	130	12.15	14.78
BW65	147.39	30.27	13.12	24.32	2.36	84.49	10.91	6.37	2.75	5.2	2.09	88.94	136.74	10.56	14.52
BW66	144.14	37.67	16.18	24.84	1.77	143.52	12.66	6.35	2.53	5.24	2.11	100	142	10.89	15.26
BW67	134.77	29.95	14.31	25	3.17	142.39	14.71	8	2.62	5.58	2.02	95	130	13.3	29.7
BW68	126.08	28.41	14.49	27.36	2.46	133.24	15.16	6.29	2.82	4.4	2.03	108	145	9.04	15.74
BW69	144.04	35.38	16.01	29.08	2.12	136.06	11.75	6.43	3.02	4.54	1.94	95.44	142.61	13.74	15.33
BW70	151.74	33.81	14.92	27.17	2.04	106.12	15.63	6.97	3.12	5.38	2.2	105	146.67	9.26	14.59
BW71	130.22	26.43	12.46	25.86	3.18	135.8	16.3	6.95	2.94	5.5	2.18	100	140	10.19	15.04
BW72	136.55	33.95	15.85	24.72	3.22	122.59	16.36	6.81	2.93	4.84	2	95	130	8.74	16.85
BW73	145.88	34.39	15.54	27.59	3.37	109.43	12.58	7.51	3.01	4.81	2.04	95	138.93	10.07	14.08
BW74	142.54	28.76	14.19	24.45	4.54	205.4	21.65	7.78	3.04	5.63	1.88	100	145	10.93	16.52
BW75	145.21	36.14	15.47	27.78	4.74	201.84	21.02	7.83	3.08	7.11	2.08	100	143.59	10.15	24.07
BW76	138.03	29.02	14.44	26.27	4.33	217.41	17.75	8.52	2.74	6.77	2.12	98.67	139.39	11.7	32.59
BW77	142.08	33.66	13.62	31.47	4.78	328.31	21.69	8.35	3.28	7.17	2.12	100	140	13.07	37.67
BW78	142.48	35.01	14.82	26	3.31	180.43	21.33	9.39	2.99	6.93	1.95	90	132	14.22	29.93
BW79	150.53	33.81	13.83	27.32	3.18	180.17	22.26	9.04	2.94	6.86	1.94	92.78	137.43	10.56	31.48
BW80	137.52	33.93	14.56	26.71	3.11	170.77	20.75	8.86	2.99	7.21	2.13	95	135	13.78	24.67
BW81	136.07	32.81	17.07	26.69	4.16	212.2	20.72	9.1	3.25	6.89	2.12	95	135.03	13.89	41.11
BW82	156.09	33.53	15.56	27.4	3.71	264.86	20.9	8.95	3.36	7.27	2.15	100	146.74	13.15	33.18
BW83	154.24	35.24	17.42	31.15	3.28	318.21	20.9	8.89	3.31	6.7	2.15	100	142.83	11.3	50.22
BW84	137.61	28.98	13.88	27.71	3.53	240.81	25.14	8.62	3.18	6.82	1.88	90	135	11.52	30.89
BW85	136.67	33.05	17.65	29.04	3.57	229.24	20.97	9.33	3.28	7.07	2.14	100	145	14.11	35.82
BW86	139.93	27.6	15.95	30.86	3.68	220.15	21.45	8.16	3.34	6.87	2.46	90	135	13.89	29.78
BW87	136.24	26.15	15.59	23.27	3.4	211.09	20.71	8.59	3.08	6.76	2.26	100	135	13.19	31.48
BW88	148.62	32.04	15.52	31.37	4.28	245.35	21.37	9.6	3.14	7.74	2	90.12	132.04	13.99	35.94
BW89	134.58	24.59	13.96	28.03	3.33	130.16	15.57	9	2.89	7	1.93	95	140	12.33	21.00
BW90	141.52	31.24	14.29	28.73	5.28	195.09	24.88	10.1	3.04	7.14	2.28	105	145	14.37	43.93
BW91	134.66	32.31	16.18	29.24	2.11	155.98	9.05	6.26	3.19	4.38	1.9	100	145	9.41	26.30
BW92	140.95	34.01	12.86	31.54	2.68	122.44	20.86	9.1	3.2	7.5	1.94	90	136	12.85	23.74
BW93	135.36	27.01	13.38	28.93	3.58	205	16.46	9.18	3.12	7.73	1.95	100	145	11.63	21.70
BW94	146.49	30.97	14.21	27.76	4.64	247.05	19.61	8.82	3.31	6.94	2.07	90	130	14.41	35.78
BW95	126.02	24.4	11.53	32.91	3.65	325.67	19.39	8.16	3.22	6.47	2.01	95	135	12.82	33.30
BW96	151.44	22.79	10.73	26.83	2.56	215.56	13.04	5.6	3.04	4.31	2.04	100	145	9.67	19.59
BW97	204.00	33.47	13.91	29.26	2.24	65.81	11.48	5.66	2.63	4.3	1.69	115	150	11.41	14.82
BW98	60.00	15.17	5.07	8.51	0.76	15.49	13.93	8.6	2.43	5.69	1.61	100.13	135	14.41	5.37
BW99	121.07	26.62	16.53	27.77	3.85	331.33	21.34	8.35	3.14	6.69	2.49	86	125	15.48	60.89
BW100	98.92	26.16	13.34	25.83	2.65	152.57	17.76	7.33	3.06	5.47	2.06	90	135	10.89	16.00
GM	141.32	31.31	14.53	27.35	3.52	183.29	20.95	8.44	3.03	6.37	2.07	97.49	138.77	12.7	28.62
Std. Deviation	15.44	4.83	1.94	3.27	0.99	65.15	5.01	1.16	0.316	0.91	0.2	7.24	6.9	2.04	10.96
Std. Error	0.48	0.15	0.06	0.1	0.03	2.03	0.16	0.04	0.01	0.03	0.01	0.23	0.22	0.06	0.34
Minimum	60	15.17	5.07	8.51	0.76	15.49	9.05	5.6	1.95	4.01	1.24	86	125	7.67	5.00
Maximum	204	47.06	18.75	33.46	5.61	387.14	29.72	10.13	3.58	8.52	2.49	127	157	21.75	61.89
CV%	10.93	15.41	13.36	11.96	28.22	35.54	23.93	13.73	10.43	14.3	9.57	7.43	4.97	16.09	38.31
P Value	***	***	***	***	***	***	***	***	***	***	***	***	***	***	***
LSD (0.05)	2.34	1.25	0.42	1.07	0.3	12.26	0.35	0.21	0.15	0.19	0.1	0.03	0.008	0.65	1.23

Abbreviations: GM, grand mean; CV, coefficient of variation; LSD, least significant difference.

*** Significant at p < 0.001.

**TABLE 3:** Mean performance of yield and yield related 15 agromorphological traits in 100 CWF RIL lines.

Rice Breeding lines	PH (cm)	FLL (cm)	FLW (mm)	PnL (cm)	PnW t (g)	GrPn	GrWt (g)	GL (mm)	GB (mm)	KL (mm)	KB (mm)	HD (days)	MT (days)	Till	PY (g)
Chakhao (control black rice)	156.55	45.51	14.37	25.67	2.89	86.82	23.28	8.76	3.12	6.68	2.09	112	153.00	8.57	14.95
Chenga (Parent 1)	148.25	32.68	12.27	32.67	2.36	127.31	22.49	8.13	3.11	6.45	2.02	110	145.39	10.57	24.79
Oryza rufipogon (Parent 2)	163.29	20.28	7.69	18.89	1.08	26.77	17.38	8.48	2.08	6.71	1.42	127	157.00	20.85	10.66
CW1	159.24	31.67	13.47	28	2.76	129.11	28.52	9.14	3.23	6.3	2.12	100.33	137.59	13.05	37.99
CW2	146.38	33.09	14.11	24.84	1.58	116.82	25.08	9.25	2.82	6.61	1.96	104.59	144.63	10.49	24.48
CW3	143.21	36.78	16.35	30.88	3.17	88.52	20.97	9.27	3.18	6.57	2.15	99.52	143.01	12.39	18.53
CW4	153.94	45.69	15.68	27.55	3.12	106.22	21.35	9.2	3.32	6.48	2.08	108.06	147.6	14.17	20.7
CW5	142.99	28.62	15.58	23.58	2.27	86.9	23.82	9.08	3.35	7.11	2.13	110	146.26	14.41	18.31
CW6	135.25	33.05	16.96	27.54	3.54	122.7	21.22	9.09	2.95	6.13	2.1	100.96	142.81	11.25	28.06
CW7	129.64	30.97	15.2	27.66	3.2	84.96	21.33	9.1	3.06	6.74	1.88	105.3	146.63	11.45	16.7
CW8	129.61	25.42	12.07	26.64	3.77	77.58	22.26	9.05	3.08	6.77	1.86	113.96	147.96	13.06	17.1
CW9	131.41	27.44	14.92	27.91	3.89	77.6	22.93	9.03	3.4	6.74	2.12	110.48	148.01	14.04	17.83
CW10	147.9	43.36	13.57	21.86	0.97	80.96	22.17	9.2	3.06	6.86	2.41	104.3	147.25	15.85	18.55
CW11	153.9	32.04	15.13	28.48	2.37	94.2	29.72	8.93	3.11	6.4	2.55	99.44	141.76	15.67	37.11
CW12	161.07	34.79	15.34	28.04	1.95	81.48	22.11	8.23	3.12	6.44	2.69	99.33	156.49	15.14	21.42
CW13	154.63	36.8	16.75	26.3	3.32	91.12	20.78	9.31	3.21	6.86	2.21	104.63	147.46	13.04	20.13
CW14	160.75	33.53	16.07	24.58	2.58	73.16	21.37	8.46	2.92	6.33	2.13	100	142.41	11.95	18.63
CW15	133.16	28.93	14.31	28.1	3.9	69.35	25.26	9.28	3.51	6.61	2.36	109.04	146.13	8.63	14.46
CW16	150.56	35.64	16.79	27.81	3.72	103.22	31.25	9.72	3.27	7.06	2.11	104.15	142.63	12.51	43.87
CW17	155.7	33.38	15.92	28.73	3.87	94.92	22.19	8.1	3.43	6.28	2.2	99.56	141.41	14.35	19.12
CW18	140.4	34.63	14.63	27.8	2.86	81.81	21.62	8.71	3.16	6.13	2.12	110.04	147.82	14.63	17.69
CW19	122.28	27.58	12.11	27.96	2.83	98.14	20.54	8.13	3.53	6.12	2.14	104.59	144.79	15.18	18.52
CW20	123.82	27.66	15.03	31.6	2.68	183.4	22.7	7.76	2.66	5.94	1.83	111.97	148.27	12.01	25.7
CW21	143.81	36.95	15.61	29.33	2.01	81.23	21.31	8.28	2.97	6.23	1.99	104.89	147.07	14.46	17.72
CW22	136.98	27.23	15.62	25.51	2.21	80.84	23.32	8.61	3.24	7.08	2.22	99.89	144.85	15.2	18.09
CW23	145.34	32.96	15.45	23.78	2.16	94.41	23.22	10.04	3.6	7.17	2.13	100	142.07	16.15	26.32
CW24	124.45	28.8	15.02	25.17	1.41	122.82	22.26	8.87	3.23	6.63	2.13	104.11	142.63	13.59	25.48
CW25	124.37	28.12	15.08	25.86	2.82	106.16	23.44	8.45	3.22	5.6	2.17	109.15	147.26	13.8	23.25
CW26	143.09	34.94	15.71	31.03	3.67	130.55	20.62	7.85	2.89	5.33	2.08	99.74	144.36	10.36	27.62
CW27	135.1	33.39	16.74	27.45	3.33	80.27	22.63	9.28	3.32	7.39	2.13	103.00	145.25	10.9	19.52
CW28	163.28	28.89	14.31	26.98	2.67	93.59	21.78	9.54	3.11	7.07	2.12	99.18	138.6	11.1	25.38
CW29	154.17	35.82	16.79	28.4	3.79	151.42	25.42	8.85	3.29	7.14	2.25	101.7	143.63	12.41	34.89
CW30	124.14	27.21	13.23	31.54	2.75	121.96	23.59	8.25	3.29	6.24	2.44	107.85	144.3	13.56	26.12
CW31	133.77	33.64	16.81	31.91	1.85	131.5	18.18	8.09	3.26	5.61	2.34	109.07	146.35	13.6	22.29
CW32	144.13	37.6	16.61	25.08	3.23	108.3	29.27	9.22	3.3	7.15	2.47	109.19	147.72	15.18	19.15
CW33	159.87	28.63	14.3	27.97	5.44	162.72	22.93	8.64	3.11	5.8	2.11	110.33	145.33	15.11	30.97
CW34	135.15	35.1	16.77	26.59	2.22	111	22.7	8.68	2.63	5.54	1.56	107.22	145.77	11.79	21.96
CW35	124.26	28.89	15.12	25.37	4.09	152.66	21.63	8.59	3.09	6.37	2.34	109.59	148.31	13.6	25.02
CW36	145	39.15	15.71	28.58	2.72	126.56	24.64	9.17	3.29	7.22	2.1	105.89	141.04	16.7	31.12
CW37	124.04	29.18	14.97	25.8	1.9	110.89	22.12	8.22	3.07	6.75	2.41	104.96	140.7	17.31	26.7
CW38	143.79	36.27	16.01	25.84	2.71	69.71	22.06	8.01	3.12	6.26	2.41	101.30	140.56	15.43	19.16
CW39	135.88	35.48	16.7	27.88	2.77	112.12	21.47	8.85	3.11	6.67	2.32	100.74	142.81	15.34	32.52
CW40	136.7	28.66	15.31	27.73	5.14	138.47	21.43	8.46	3.32	5.85	2.36	105.15	145.99	15.15	34.6
CW41	158.34	30.75	12.13	25.93	3.04	139.63	25.5	9.24	3.53	5.85	2.63	110.19	145.44	15.1	32.01
CW42	136	33.45	16.93	27.88	3.41	60.74	24.41	9.21	3.31	7.15	2.11	112.11	150.59	16.26	16.96
CW43	125.1	27.96	15.2	27.14	3.33	74.25	28.07	8.5	3.31	6.84	2.31	114.93	148.59	15.2	19.62
CW44	144.74	37.37	16.6	36.61	3.72	142.43	19.26	6.91	3.13	5.04	2.35	106.56	143.2	13.12	31.5
CW45	162.31	27.45	14.5	25.23	2.72	74.59	24.45	9.3	3.22	6.84	2.32	108.07	146.74	16.08	19.6
CW46	134.35	33.26	16.48	27.85	3.09	94.53	21.99	8.96	3.14	5.94	2.15	109.37	145.99	13.95	14.51
CW47	144.26	35.98	16.54	26.81	2.36	81.52	20.3	8.46	3.16	6.56	2.33	100.22	139.44	14.57	11.23
CW48	152.65	36.92	16.88	22.93	2.03	100.89	21.63	7.13	3.12	5.13	2.16	100.52	138.74	16.06	16.23
CW49	123.63	28.75	13.39	26.14	2.85	103.59	21.67	9.12	3.05	6.23	2.26	98.75	141.15	16.54	20.39
CW50	143.59	34.71	15.15	25.99	2.93	118.93	14.57	8.6	3.09	6.89	2.22	103.93	144.52	14.97	18.06
CW51	124.07	28.03	13.35	27.27	3.45	83.89	15.93	8.39	2.91	6.34	2.16	110.11	147.48	14.91	14.91
CW52	123.59	28.74	13.28	24.91	2.43	78.52	17.33	9.28	3.13	6.79	2.13	100.11	141.79	13.02	15.38
CW53	153.34	35.19	15.45	24.98	2.58	98.84	17.49	7.86	3.18	6.34	2.35	103.04	143.26	12.8	21.75
CW54	144.33	32.1	14.4	25.42	2.8	86.19	18.24	9.09	2.94	6.81	2.55	111.56	144.78	14.36	18.78
CW55	143.96	26.93	15.32	30.79	3.24	100.78	18.03	8.85	2.96	6.7	2.54	113.85	148.04	13.37	23.9
CW56	143.74	33.97	14.16	31.19	3.94	94.47	18.17	8.88	3.02	6.66	2.44	102.05	142.61	14.19	24.74
CW57	133.23	31.66	15.29	31.73	6.09	110.84	18.34	9.13	3.18	7.14	2.55	102.78	142.48	12.53	24.36
CW58	153.83	12.8	14.1	24.87	2.04	90.96	18.75	8.95	3.11	7.15	2.29	108.52	144.37	13.38	20.36
CW59	155.65	34.07	16.92	25.08	2.29	84.89	19.5	9.08	3.11	7.02	2.29	99.37	141.96	14.46	15.6
CW60	146.39	34.37	16.05	25.46	2.33	123.96	19.37	8.66	2.68	6.15	2.11	99.78	138.44	14.93	23.75
CW61	134.93	24.48	11.42	27.03	2.58	73.91	19.49	9.1	3.1	6.76	2.13	104.67	142.72	14.49	14.69
CW62	164	33.56	16.09	25.86	2.34	74.93	20.58	9.09	3.03	6.72	2.24	99.96	143.02	13.09	16.96
CW63	170.53	26.1	13.07	27.77	2.25	85.93	20.87	9.13	3.07	6.64	2.16	108.63	147.02	13.52	15.45
CW64	124.43	26.6	14.4	25.78	1.73	85.22	21.22	9.01	2.94	6.35	2.52	105.04	144.94	13.77	15.33
CW65	140.86	28.8	15.19	27.6	1.96	83.41	21.17	9.35	3.1	7.09	2.11	109.15	148.82	11.83	14.88
CW66	154.78	35.08	15.78	25.08	2.09	85.95	21.23	9.33	3.12	6.17	2.17	104.21	141.81	11.33	14.3
CW67	145.43	26.94	17.86	25.78	2.54	73.93	21.19	9.03	3.12	7.15	2.31	108.96	147.35	12.72	14.86
CW68	132.68	32.7	15.18	25.31	2.43	90.98	21.26	9.12	3.13	6.71	2.28	99.81	141.15	14.3	15.16
CW69	126.96	26.12	11.72	26.55	2.43	115.62	21.68	8.55	2.99	6.32	2.2	100.00	140.96	13.96	27.86
CW70	134.9	33.47	17.05	25	2.47	72.35	21.87	8.56	2.97	6.32	2.15	108.96	147.03	14.6	13.91
CW71	143.39	27.39	15.88	25.22	2.28	81.52	21.93	9.54	3.22	7.36	2.12	105.41	148.3	14.67	14.59
CW72	163.1	32.2	14.21	24.87	2.31	80.46	22.2	8.87	3.15	6.87	2.3	111.07	153.33	13	15.98
CW73	144.85	31.77	14.27	28.37	4.52	120.38	22.23	8.8	3.2	6.84	2.37	112.26	152.66	12.56	31.3
CW74	142.5	36.64	16.02	31.19	3.21	74.76	22.64	8.58	3.13	6.95	2.33	110.19	148.38	12.95	14.85
CW75	155.21	35.86	16.17	27.92	1.79	84.58	23.2	8.45	2.79	6.3	2.33	104.48	145.74	14.82	14.35
CW76	154.2	33.94	15.62	28.69	2.5	68.45	23.04	8.14	3.15	6.38	2.12	100.96	143.63	13.53	15.67
CW77	124.07	26.55	13.08	32.08	3.07	86.31	23.27	8.96	3.43	7.13	2.13	110.85	145.93	14.67	18.19
CW78	124.8	26.16	14.45	28.59	3.32	99.78	23.23	8.79	3.34	6.74	2.17	110.30	146.09	14.83	32.44
CW79	131.28	29.05	14.37	26.18	3.45	132.19	23.48	8.3	3.22	6.12	2.29	111.37	149.89	14.3	37.76
CW80	125.49	26.43	12.28	26.63	2.4	109.71	23.86	8.25	3.14	6.11	2.14	113.02	152.05	13.46	29.11
CW81	133.94	34.65	16.92	27.65	4.1	153.5	23.73	9.81	3.67	7.14	2.51	99.93	139.26	12.9	25.97
CW82	143.25	35.68	16.33	26.08	3.19	145.87	23.7	8.49	3.11	7.11	2.12	104.26	144.91	11.82	29.54
CW83	135.5	28.92	14.94	24.95	2.81	103.59	23.63	8.87	3.17	6.84	2.13	108.93	145.85	12.74	34.19
CW84	155.3	35.03	15.67	31.82	2.29	119.99	23.86	9.88	3.26	6.88	2.12	99.85	141	20.78	36.08
CW85	154.28	35.47	16.85	31.43	2.55	111.17	24.18	9.93	2.9	7.02	2.14	99.85	139.58	11.43	31.55
CW86	132.18	31.58	14.8	30.73	2.64	109.21	24.48	8.77	2.9	6.78	2.14	107.33	148.9	22.33	25.04
CW87	144.46	35.63	16.45	28.71	1.88	75.77	24.73	9.06	3.13	6.43	2.11	99.78	139.67	14.35	21.19
CW88	154.97	36.23	15.74	19.06	1.55	83.42	24.67	9.45	3.23	6.32	2.13	110.85	144.52	14.53	21.63
CW89	154.59	34.58	16.17	24.89	2.92	85.11	24.63	9.12	3.08	6.48	2.12	111.22	147.18	12.61	22.57
CW90	124.77	25.41	11.48	25.4	2.91	95.99	24.73	9.27	3.44	6.11	2.13	95.00	135.44	13.16	33.65
CW91	134.46	31.96	14.78	25.07	2.71	84.89	24.81	8.65	3.23	6.54	2.14	99.85	137.7	12.42	32.44
CW92	153.46	32.12	15.71	25.38	2.46	63.83	24.74	8.87	3.2	5.78	2.14	104.33	142.37	13.91	24.58
CW93	162.88	33.65	15.69	28.31	5.13	95.78	25.35	8.91	3.23	5.61	2.32	101.15	141.81	15.02	34.75
CW94	124.81	26.35	12.13	28.51	3.47	87.77	25.7	8.71	3.26	6.11	2.22	100.20	145.57	14.66	29.62
CW95	132.18	30.75	15.05	27.94	2.73	134.54	25.98	9.28	3.22	7.11	1.99	96.59	137.41	14.36	22.78
CW96	145.02	33.19	14.53	26.18	2.72	156.84	26.56	8.82	3.19	5.54	2.08	100.85	140.81	14.13	34.95
CW97	134.13	33.24	17.03	31.24	3.75	159.41	26.65	8.67	3.15	6.13	2.1	104.62	141.58	14.39	25.5
CW98	134.65	31.52	15.17	33.14	5.42	141.12	27.7	8.73	2.86	5.85	2.12	111.91	145.65	11.33	23.44
CW99	151.97	34.98	13.25	28.41	1.96	82.56	28.19	8.29	3.13	6.13	2.56	104.74	142.06	21.8	21.05
CW100	163.15	34.69	15.85	26.79	3.49	82.68	29.99	8.64	2.69	6.22	2.41	107.64	144.97	14.61	15.57
GM	142.46	31.9	15.02	27.23	2.88	100.6	22.67	8.82	3.13	6.51	2.21	105.51	144.73	13.97	22.80
Std. Deviation	12.42066	4.76843	1.61231	2.72088	0.89194	26.83416	2.94839	0.52574	0.21374	0.50137	0.19065	5.21242	4.04948	2.62309	7.16973
Std. Error	0.38701	0.14858	0.05024	0.08478	0.02779	0.83612	0.09187	0.01638	0.00666	0.01562	0.00594	0.16241	0.12618	0.08173	0.2234
Minimum	122.28	12.8	7.7	18.75	0.97	26.8	14.57	6.9	2.08	5.04	1.42	95	135	8.54	10.66
Maximum	170.53	45.69	17.86	36.61	6.09	183.4	31.25	10.04	3.67	7.39	2.69	127	157.93	22.33	43.87
CV%	8.72	14.95	10.73	9.99	30.98	26.67	13.01	5.96	6.82	7.71	8.64	4.94	2.8	18.77	31.44
P Value <0.001	***	***	***	***	***	***	***	***	***	***	***	***	***	***	***
LSD (0.05)	1.502	1.198	0.384	0.847	0.076	5.499	0.369	0.044	0.032	0.036	0.028	0.989	1.213	1.338	1.386

Abbreviations: GM, grand mean; CV, coefficient of variation; LSD, least significant difference.

*** Significant at p < 0.001.

### Genetic variability of the 15 yield-contributing traits with broad-sense heritability

The genetic parameters pertaining to the degree of variability among the RIL genotypes (BWF and CWF) were estimated (Table 4) using the GCV, PCV, H%, GA, and GAM. For the majority of the characters, the magnitude of PCV was significantly greater than that of GCV, indicating the role of genetic factors for trait development. Differences between GCV and PCV were less in both the RIL populations (BWF and CWF), indicating a higher correlation between phenotype and genotype, less environmental effect, and a larger role of genetic factors in these traits’ expression (Table 4). H% was high (>80%) in all the characters studied, except FLL (74.42%), which indicates little environmental influence. The H% for traits ranged from 74.42% (FLL) to 98.01% (GrWt) in BWF lines and from 76.58% (GB) to 98.71% (GrPn) in CWF lines. H% was found to be more than 90% high in eleven traits out of fifteen, such as PH, PnWt, GrPn, GL, GB, KL, KB, GrWt, HD, MT, and PY, in BWF population. Whereas in CWF population, H% more than 90% was recorded for the following traits: FLW, PnWt, GrPn, GrWt, GL, KL, and KB (Table 4). High heritability (>80%) in combination with high GA (>20) was observed for the following traits: PH, PnL, GrPn, GrWt, and PY in the BWF population and PH, PnL, GrPn, and KB in the CWF population, suggesting additive gene action for the characteristics. It was observed that PnWt, GrPn, GrWt, and Till had high GAM (>40%) in the BWF RIL and PnWt, GrPn, and PY in the CWF RIL population.

**TABLE 4:** Descriptive statistics and broad-sense heritability (H%) for 15 traits in both the RIL populations (BWF and CWF).

BWF RIL population of 100 genotypes									
Traits	Mean ± SE	Range	GV	PV	GCV (%)	PCV (%)	H (%)	GA	GAM
PH (cm)	141.32 ± 0.48	60.00–204.00	115.13	125.76	7.62	7.96	91.55	24.17	17.16
FLL (cm)	31.31 ± 6.15	15.17–47.06	13.05	17.54	11.56	13.4	74.42	10.01	32.05
FLW (mm)	14.53 ± 0.06	5.07–18.75	1.69	2.07	8.89	9.83	81.66	3.28	22.44
PnL (cm)	27.35 ± 0.10	8.51–33.46	4.29	5.86	7.55	8.82	87.25	20.84	21.27
PnWt (g)	3.52 ± 0.03	0.76–5.61	0.64	0.69	22.49	23.45	91.98	11.79	50.43
GrPn	183.29 ± 2.03	15.49–387.14	2,657.89	3,287.32	27.87	28.06	90.85	89.54	71.09
GrWt (g)	20.95 ± 0.16	9.05–29.72	20.61	20.95	21.58	21.75	98.01	22.51	45.23
GL (mm)	8.44 ± 0.04	5.60–10.13	1.04	1.09	12.08	12.34	95.76	13.87	26.01
GB (mm)	3.03 ± 0.01	1.95–3.58	0.057	0.059	7.8	7.93	96.61	4.51	16.65
KL (mm)	6.37 ± 0.03	4.01–8.52	0.661	0.677	12.68	12.83	97.46	1.71	26.77
KB(mm)	2.07 ± 0.01	1.24–2.49	0.016	0.017	6.15	6.22	97.82	2.26	12.97
HD (days)	97.49 ± 0.23	86.00–127.00	35.27	35.32	6.13	8.13	98.27	12.26	12.65
MT (days)	138.77 ± 0.22	125.00–157.00	37	37.45	4.4	4.42	97.81	12.7	9.18
Till	12.7 ± 0.06	7.67–21.75	2.5	3.81	12.48	15.4	85.68	19.96	40.02
PY(g)	28.62 ± 0.34	5.00–61.89	90.16	100.17	32.74	32.95	90.01	21.76	75.03

CWF RIL population of 100 genotypes									
Traits	Mean ± SE	Range	GV	PV	GCV%	PCV%	H (%)	GA	GAM
PH(cm)	142.46 ± 0.39	122.28–170.53	128.71	145.17	8.03	8.53	88.66	26.39	18.69
FLL (cm)	31.9 ± 0.15	12.80–45.69	16.3	18.78	12.72	13.66	86.78	9.59	30.24
FLW (mm)	15.02 ± 0.05	7.70–17.86	1.6	1.71	8.38	8.67	93.49	2.79	18.49
PnL (cm)	27.23 ± 0.08	18.75–36.61	5.32	6.72	8.49	9.55	79.14	23.01	22.13
PnWt (g)	2.88 ± 0.03	0.97–6.09	0.58	0.69	27.1	29.65	91.54	10.87	66.93
GrPn	100.6 ± 0.84	26.80–183.40	416.52	597.11	21.23	25.42	98.79	60.35	62.77
GrWt (g)	22.67 ± 0.09	14.57–31.25	7.28	7.42	11.88	11.99	93.39	15.67	24.96
GL (mm)	8.82 ± 0.02	6.90–10.04	0.27	0.272	5.89	5.91	95.34	1.08	12.23
GB (mm)	3.13 ± 0.01	2.08–3.67	0.033	0.034	5.81	5.87	76.58	7.38	12.25
KL (mm)	6.51 ± 0.02	5.04–7.39	0.221	0.222	7.24	7.25	94.12	5.97	14.97
KB(mm)	2.21 ± 0.01	1.42–2.69	0.0283	0.0287	7.6	7.64	91.07	70.35	15.86
HD (days)	105.51 ± 0.16	95.00–127.00	18.77	19.43	4.12	4.19	89.11	9.25	8.79
MT (days)	144.73 ± 0.13	135.00–157.93	11.17	11.5	2.31	2.35	77.12	7.1	4.91
Till	13.97 ± 0.08	8.54–22.33	3.48	5.08	13.12	15.85	84.67	15.62	39.52
PY (g)	22.8 ± 0.22	10.66–43.87	33.22	46.24	26.27	31.00	86.71	16.55	75.44

### Trait correlation

**FIGURE 2:**
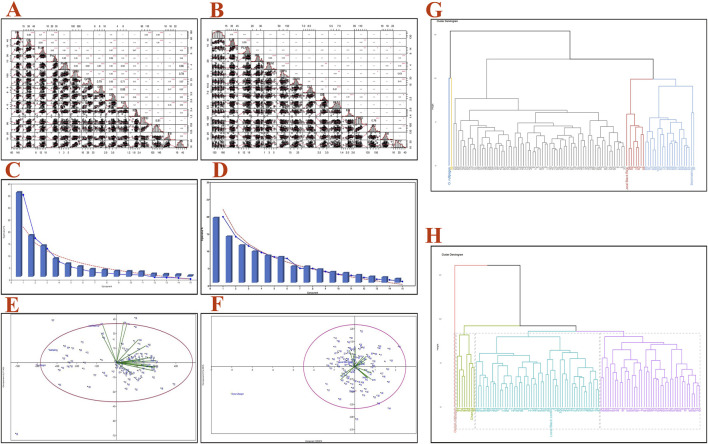
Pearson’s correlations, PCA bi-plot, and clustering dendrogram for both the RIL populations (BWF and CWF) based on 15 agro-morphological traits. Pearson’s correlation coefficient matrix of the 15 agro-morphological traits of 100 BWF RILs **(A)** and 100 CWF RILs **(B)**. Scree plot showing eigenvalue % and components of the RIL populations BWF **(C)** and CWF **(D)**. PCA bi-plot distribution of 100 RILs and 15 quantitative traits across the first two components based on PCA scores of BWF **(E)** and CWF **(F)**. Clustering dendrogram of the two RIL populations using 15 traits showing five clusters in BWF **(G)** and four clusters in CWF **(H)**, where different colors and heights of the clusters tree indicate the grouping of the genotypes into different main clusters. Plant height (PH), flag leaf length (FFL), flag leaf width (FLW), panicle length (PnL), panicle weight (PnWt), grain per panicle (GrPn), grain length (GL), grain breadth (GB), kernel length (KL), kernel breadth (KB), 1,000 grain weight (GrWt), tiller number (Till), heading date (HD), maturity time in days (MT), and single plant yield (PY).

The correlation coefficients among 15 traits for both the RIL genotypes (100 lines) are shown in Figures 2A, B. GrPn, PnWt, GrWt, and GL were positively correlated with YP in BWF, and PnL, PnWt, GrPn, and GB were positively correlated with YP in CWF (Figures 2A, B). In contrast, HD and MT were negatively correlated with YP in BWF, and KL, HD, and MT were negatively correlated with YP in CWF. The highest value of positive correlation was observed (0.88) between the traits KL and GL, followed by GL and GrWt (0.79), GL, and PnWt (0.61). HD was negatively correlated with three traits: GrWt (−0.22), GB (−0.0.23), and KB (−0.03) in BWF. The highest positive correlation (0.76) was found between HD and MT and between GL and KL (0.61) in CWF. PY was positively correlated with GrPn and PnWt in both the RILs (BWF and CWF) while being negatively correlated with HD and MT. The correlation analysis, therefore, indicates that GrPn, PnL, PnWt, GrWt, and GL are the most important traits that need to be considered in the production of high-yield breeding lines.

### Cluster analysis and dendrogram construction

The UPGMA dendrogram grouped 100 BWF RILs into 5 clusters and 100 CWF RILs into 4 clusters (Figures 2C, D). The cluster means for 15 traits among 100 BWF RILs and 100 CWF RILs are presented in Table 5. The BWF dendrogram cluster I consists of one genotype of only wild rice (blue) and is grouped separately, whereas cluster II contains a single genotype of the shortest PH of only 60 cm (yellow); clusters III, IV, and V were polygenotypic, comprising 72 genotypes, eight genotypes, and 24 genotypes, respectively (gray, purple, and greenish colors). Similarly, 100 genotypes of CWF RIL population were grouped into four clusters: I, II, III, and IV, with 1, 8, 52, and 42 genotypes, respectively (different colors in the dendrogram).

**TABLE 5:** Cluster means for 15 traits among 100 BWF and 100 CWF RILs.

Variable	Cluster of 100 BWF lines					Cluster of 100 CWF lines			
	I	II	III	IV	V	I	II	III	IV
PH	163.29	60	138.22	163.11	139.93	163.292	139.51	141.82	143.31
FLL	20.28	15.17	30.9	37.86	31.04	20.278	33.73	31.53	32.29
FLW	7.69	5.07	14.73	14.68	14.05	7.688	15.65	15.05	15.05
PnL	18.89	8.51	27.88	27.68	25.99	18.889	30.42	26.6	27.61
PnWt	1.08	0.76	3.86	4.03	2.41	1.083	2.99	2.81	2.98
Gr/Pn	26.77	15.49	211.51	164.16	140.99	26.77	132.78	88.49	111.23
GrWt	17.38	13.93	22.69	22.17	12.95	17.378	21.91	22.15	23.58
GL	8.48	8.6	8.88	8.99	6.49	8.483	7.91	8.83	8.98
GB	2.08	2.43	3.15	3.07	2.75	2.083	2.96	3.15	3.17
KL	6.71	5.69	6.76	6.77	4.89	6.706	5.62	6.65	6.49
KB	1.42	1.61	2.09	2.06	2.01	1.421	2.06	2.23	2.22
HD	127	100.13	95.92	110.75	98.86	127	107.12	107.56	102.15
MT	157	135	137.07	151.04	140.27	157	144.71	146.73	141.97
Till	20.85	14.41	13.05	13.45	10.67	20.852	12.36	13.79	14.34
PY	10.66	5.37	33.66	27.34	16.6	10.658	24.19	19.16	27.33

Mahalanobis D^2^ test for genetic diversity assessment in BWF and CWF.

Mahalanobis D^2^ statistic is widely used to analyze the relative contribution of various yield components to total divergence, and it also classifies different genotypes into suitable clusters based on their genetic distances (D^2^ values) following Tocher’s method. Mahalanobis D^2^ statistic estimates the relative contribution of several components at the intra- and intercluster levels, and genotypes derived from widely divergent clusters are likely to form heterotic combinations. The 100 genotypes of BWF RIL populations were grouped into seven clusters following Tocher’s method based on Mahalanobis D^2^ distance values (Tables 6 and 7). Among them, five clusters are polygenotypic (i.e., I, IV, V, VI, and VII), whereas clusters II and III were monogenotypic and predicted uniqueness in the genes (Supplementary Table S3). The average intra- and intercluster D^2^ distance values are represented in Table 6. Intra-cluster D^2^ values ranged from 0.00 (clusters II and III) to 2,233.83 (cluster VI), followed by clusters IV (1,688.55), V (1,567.85), VII (1,299.42), and I (952.77). The highest intra-cluster distance (2,233.83) in cluster VI indicates wide genetic variation among the genotypes belonging to these clusters, and cluster I recorded the lowest intra-cluster distance (952.77), suggesting a closer relationship and low degree of diversity among the genotypes of this cluster. Clusters II and III consisted of only one genotype each; hence, they lacked intra-cluster distance (0.00). The largest intercluster distances were found between clusters IV and VII (25,865.50) in the BWF lines, indicating that genotypes in cluster IV were far diverse from those of VII. The least distance was observed between clusters I and II (4,047.36), which indicated that genotypes included in these cluster were closely related (Table 6). The cluster-wise mean values for the 15 characters in BWF are presented in Table 7. These are helpful to assess the superiority of the clusters during the improvement of characters through a hybridization program. The cluster mean values showed a wide range of variation for the majority of the characters undertaken in the present study. It was observed that cluster II had recorded the highest mean values for most of the traits, followed by clusters IV, VI, and VII (Table 7). The contribution of different traits to total divergence is depicted in Table 7. The trait GrPn (19.00) showed the maximum contribution toward genetic divergence, followed by GrWt (13.90), KB (13.60), GB (10.50), and PH (8.50). Out of the 15 agro-morphological traits studied, only five traits (GrPn, GrWt, KB, GB, and PH) provided the maximum contribution (65.50%) toward total divergence (Table 7). On the other hand, based on the D^2^ matrix, 100 CWF RILs were grouped into 11 Tocher’s clusters, of which seven were multi-genotypic and four were mono-genotypic (Supplementary Table S4). Each of the 15 traits that contributed to the overall genetic divergence in the CWF was categorized and displayed in Table 8. The contribution toward the total variation was the maximum for GrWt (24.32), followed by the other traits (Table 8). Cluster XI had the highest PY (34.95 g), with the maximum contributions from GrWt, GL, KB, PH, GB, PnL, GrPn, and PnWt. Moreover, PY benefited most from clusters VIII (32.01 g) and X (33.65 g) (Table 9). The average intra- and intercluster distances within the 11 clusters indicate the degree of divergence within and between the groups (Tables 8 and 9). The largest intercluster distances (25,817.49) were found between cluster II (CW27, CW71, CW95, CW23, CW16, CW84, CW85, and CW81) and cluster IV (CW44, CW48, CW26, and CW31), containing genotypes that were found to be the most divergent with the maximum intercluster distance. According to the D^2^ cluster matrix, cluster VII had the largest intra-cluster distance (2942.63) with RIL genotypes CW20 and CW34. The maximum heterosis would result from a cross between genotypes from clusters II and IV, which had the greatest genetic distance (25,817.49).

**TABLE 6:** Average intercluster and intra-cluster distances (D^2^) among the seven clusters of BWF.

	Cluster I	Cluster II	Cluster III	Cluster IV	Cluster V	Cluster VI	Cluster VII
Cluster I	**952.7771**	4,047.366	5,718.752	24,521.49	16,088.54	12,355.73	7,650.93
Cluster II	4,047.3664	**0**	8,242.971	6,877.44	8,434.66	7,300.83	5,308.49
Cluster III	5,718.7515	8,242.971	**0**	7,877.31	10,258.63	6,866.99	9,955.35
Cluster IV	24,521.49	6,877.44	7,877.31	**1,688.55**	19,653.73	17,816.69	25,865.5
Cluster V	16,088.54	8,434.66	10,258.63	19,653.73	**1,567.85**	12,534.64	23,744.32
Cluster VI	12,355.73	7,300.83	6,866.99	17,816.69	12,534.64	**2,233.83**	9,278.97
Cluster VII	7,650.93	5,308.49	9,955.35	25,865.5	23,744.32	9,278.97	**1,299.42**

Intra- (bold) and intercluster distances.

**TABLE 7:** Cluster mean values estimated by Tocher’s method from 100 BWF RIL populations and the percent contribution of each trait toward total divergence.

Clusters	I	II	III	IV	V	VI	VII	% contribution to the total variation
Traits								
PH	135.25	204.00	60.00	128.20	157.87	144.04	135.77	8.50
FLL	29.99	33.47	15.17	28.05	34.42	32.68	30.36	1.40
FLW	14.92	13.91	5.07	13.26	14.59	15.02	15.08	2.30
PnL	27.32	29.26	8.51	26.78	27.21	28.51	27.46	1.70
PnWt	3.44	2.24	0.76	3.67	3.51	3.75	3.66	5.30
GrPn	188.97	65.81	15.49	213.35	178.14	187.3	180	19.00
GrWt	19.48	11.48	13.93	19.95	21.63	21.55	24.18	13.90
GL	8.16	5.66	8.60	8.32	8.58	8.57	8.89	8.80
GB	3.00	2.63	2.43	3.01	3.09	3.08	3.13	10.50
KL	6.27	4.30	5.69	6.17	6.48	6.52	6.50	5.00
KB	2.08	1.69	1.61	2.15	2.13	2.08	2.07	13.60
HD	95.64	115.00	100.13	96.91	101.89	95.67	94.63	2.10
MT	136.46	150.00	135.00	134.36	144.88	138.2	136.92	2.50
Till	12.41	11.41	14.41	12.13	12.89	12.49	13.49	1.10
PY	27.48	14.82	5.37	32.45	28.71	29.92	30.17	4.30
No. of traits with the highest mean	0	4	1	3	1	3	3	

**TABLE 8:** Average intercluster and intra-cluster distances (D^2^) among the eleven clusters of CWF.

	Cluster I	Cluster II	Cluster III	Cluster IV	Cluster V	Cluster VI	Cluster VII	Cluster VIII	Cluster IX	Cluster X	Cluster XI
Cluster I	**2,092.17**	3,671.324	4,654.261	15,520.32	4,342.561	4,543.374	9,910.027	8,255.03	3,380.248	4,894.26	9,000.962
Cluster II	3,671.324	**1,817.28**	9,498.773	25,817.49	8,147.964	10,042.14	16,859.5	12,948.33	3,423.506	6,741.135	14,614.04
Cluster III	4,654.261	9,498.773	**2,186.38**	6,874.335	3,047.787	3,911.667	4,739.856	4,148.821	8,968.021	3,674.098	2,946.769
Cluster IV	15,520.32	25,817.49	6,874.335	**2,282.17**	10,206.61	8,600.789	5,961.776	9,178.479	22,636.23	12,249.61	5,117.101
Cluster V	4,342.561	8,147.964	3,047.787	10,206.61	**1,445.49**	3,983.65	7,347.193	4,253.258	7,732.891	4,855.543	4,571.699
Cluster VI	4,543.374	10,042.14	3,911.667	8,600.789	3,983.65	**2,732.27**	8,839.519	6,627.903	6,291.942	7,508.677	7,817.698
Cluster VII	9,910.027	16,859.5	4,739.856	5,961.776	7,347.193	8,839.519	**2,942.63**	10,235.98	18,238.08	8,523.781	3,813.556
Cluster VIII	8,255.03	12,948.33	4,148.821	9,178.479	4,253.258	6,627.903	10,235.98	**0**	11,804.11	4,043.698	3,185.006
Cluster IX	3,380.248	3,423.506	8,968.021	22,636.23	7,732.891	6,291.942	18,238.08	11,804.11	**0**	8,351.576	16,070.24
Cluster X	4,894.26	6,741.135	3,674.098	12,249.61	4,855.543	7,508.677	8,523.781	4,043.698	8,351.576	**0**	3,332.677
Cluster XI	9,000.962	14,614.04	2,946.769	5,117.101	4,571.699	7,817.698	3,813.556	3,185.006	16,070.24	3,332.677	**0**

Intra- (bold) and intercluster distances.

**TABLE 9:** Cluster mean values estimated by Tocher’s method from 100 CWF RIL populations and the percent contribution of each trait toward total divergence.

Cluster	I	II	III	IV	V	VI	VII	VIII	IX	X	XI	% contribution to the total variation
PH	141.4	143.76	141.4	143.56	158.52	157.21	129.48	158.34	133.23	124.77	145.02	7.43
FLL	31.37	33.16	31.85	35.72	33.37	34.99	31.38	30.75	31.66	25.41	33.19	1.77
FLW	15.06	16.17	14.91	16.5	15.49	15.39	15.9	12.13	15.29	11.48	14.53	3.55
PnL	26.81	27.89	27.55	29.83	27.64	26.51	29.09	25.93	31.73	25.4	26.18	5.79
PnWt	2.77	2.9	3.16	2.82	2.93	2.27	2.45	3.04	6.04	2.91	2.72	4.97
GrPn	93.45	109.83	105.42	126.34	88.44	90.16	144.23	139.63	110.84	95.99	156.84	5.83
GrWt	22.17	24.54	23.24	19.92	29.86	19.8	22.7	25.5	18.34	24.73	26.56	24.32
GL	8.93	9.68	8.61	7.5	8.79	8.05	8.22	9.24	9.13	9.27	8.82	13.1
GB	3.14	3.31	3.13	3.1	2.9	3.15	2.65	3.53	3.18	3.44	3.19	7.09
KL	6.76	7.14	6.08	5.29	6.31	6.39	5.74	5.85	7.143	6.11	5.54	3.69
KB	2.22	2.16	2.2	2.23	2.48	2.52	1.7	2.63	2.55	2.13	2.08	10.69
HD	105.86	101.1	105.77	103.97	103.54	101.18	109.6	110.19	102.78	96.19	100.85	2.33
MT	145.08	141.94	144.4	143.16	143.36	149.88	147.02	145.44	142.48	135.44	140.81	2.1
Till	13.89	14.21	14.37	13.29	15.14	13.97	11.9	15.1	12.53	13.16	14.13	2.12
PY	20.96	27.33	24.1	24.41	26.34	21.59	23.83	32.01	24.36	33.65	34.95	5.22
No. of traits with the highest mean	0	1	0	2	3	1	0	3	3	0	2	

#### Principal component analysis (PCA)

To find out the independent impact of all the traits under study and to reveal the patterns of genetic variation among the rice RIL genotypes, PCA was conducted. The visual scree plot (Figures 2C, D) showed the amount of variance described by each PC. The results of the PCA showed that the first four PCs contribute 73.74% of the cumulative variability (PC1, 35.52%; PC2, 17.43%; PC3, 13.09%; and PC4, 7.68%) with eigenvalues >1, indicating significant variability in the BWF RILs (Table 10). PC1 has a high component loading value for PnWt (0.350), GrWt (0.346), GL (0.335), GB (0.324), KL (0.332), PY (0.356), and others, and that accounted for 35.52% of the total variation as a whole in BWF (Table 10). The main contributing variables to the first four PCs (73.74% of the cumulative variability) were PnWt, GrWt, GL, GB, KL, and PY, and these are the major drivers of differences among 100 genotypes of the BWF population. PC2 contributed 17.43%, PC3 contributed 13.099%, and PC4 contributed 7.683% of the total variability in BWF lines (Table 10). The PCA bi-plot shows relationships among genotypes, traits, and environments in a simplified manner. The PCA-bi-plot analysis indicated the comparative genetic distance between different genotypes and phenotypic characteristics by employing the first two PCs along the X- and Y-axes (Figure 2E). The distribution of genotypes based on their genetic diversity has been observed in the four quadrants of the bi-plot (Figure 2E). The RILs (BW9, BW19, BW81, BW44, BW86, and BW88) projected on the PCA bi-plot vectors of GrPn, grain weight, GL, KL and PY were close to them, demonstrating a positive interaction (Figure 2). The PCA analysis of the yield and yield-contributing traits of 100 CWF RILs generated six PCs, and the first six components together explained more than 71.90% of the total variation in CWF RILs (Figure 2; Table 11). PC1, PC2, PC3, PC4, PC5, and PC6 accounted for 20.026, 14.267, 11.569, 9.680, 8.394, and 7.961%, respectively, of the total variability in CWF RILs. The PCA scores for 100 CWF RIL genotypes in the first two PCs were estimated and plotted on a bi-plot. The distribution of genotypes based on their diversity can be observed in the four quadrants of the bi-plot (Figure 2F). Comparing the 100 CWF RILs based on the PCA bi-plot analysis, the RILs CW1, CW11, CW16, CW26, CW29, CW32, CW36, CW40, CW41, CW44, CW57, CW79, CW81, CW84, CW85, CW93, CW96, and CW98 were superior for PnWt, PnL, GrWt, PY, GL, GB, and KL (Figure 2; Table 11). The BWF RIL genotypes with a high positive principal component score for PC 1 were BW5 (1.912), BW6 (1.762), BW7 (1.577), BW8 (1.142), BW12 (1.950), BW15 (1.634), BW17 (1.935), BW18 (2.162), BW23 (4.285), BW24 (2.965), BW25 (2.786), BW30 (1.419), BW31 (1.625), BW44 (2.215), BW52 (2.737), BW77 (2.411), BW88 (2.564), BW90 (2.511), BW99 (3.362), and BW86 (1.950), and they were superior for the traits PnWt, GrWt, GL, GB, KL, and PY.

**TABLE 10:** Contribution of different traits toward the total variance in 100 BWF RIL populations (eigenvalue, contribution of variability, and factor loadings of PCs).

Eigenvalue, contribution of variability, and factor loadings for the principal component axes				
Parameter	Principal component (PC)			
	1	2	3	4
Eigenvalues	5.32865	2.61453	1.9648	1.15259
Variability (%)	35.524	17.43	13.099	7.6839
Cumulative variability (%)	35.52	52.95	66.05	73.74
Traits	Factor loadings after varimax rotation			
PH	0.063856	0.49889	0.12589	0.038321
FLL	0.094833	0.4835	−0.12153	−0.19963
FLW	0.1973	0.28426	−0.35416	−0.22389
PnL	0.26408	0.20689	−0.17599	0.31373
PnWt	0.35007	0.024973	0.042357	0.15911
GrPn	0.27336	−0.021009	−0.11571	0.60982
GrWt	0.3465	−0.061184	0.18121	−0.27597
GL	0.33538	−0.099389	0.3256	−0.22077
GB	0.32434	0.065882	−0.14066	−0.1153
KL	0.33299	−0.052085	0.28945	−0.22249
KB	0.21885	0.032489	−0.3264	−0.29149
HD	−0.13848	0.36523	0.40193	0.14283
MT	−0.12429	0.46333	0.27479	0.044102
Till	0.1518	−0.1422	0.45996	0.023818
PY	0.35611	−0.051327	0.014904	0.34259

**TABLE 11:** Contribution of different traits toward the total variance in 100 CWF RIL populations (eigenvalue, contribution of variability, and factor loadings of PCs).

Eigen value, contribution of variability, and factor loadings for the principal component axes						
Parameters	Principal component (PC)					
	1	2	3	4	5	6
Eigenvalues	3.00397	2.13998	1.73541	1.45202	1.25909	1.1942
Variability (%)	20.026	14.267	11.569	9.68	8.394	7.961
Cumulative variability (%)	20.03	34.29	45.86	55.54	63.94	71.9
Traits	Factor loadings after varimax rotation					
PH	−0.035	0.295	−0.302	0.135	0.439	0.024
FLL	0.207	0.247	−0.435	0.289	0.177	0.008
FLW	0.267	0.243	−0.289	0.360	−0.085	−0.038
PnL	0.299	−0.275	0.024	0.232	−0.132	0.040
PnWt	0.292	−0.253	0.216	0.352	−0.028	−0.006
GrPn	0.384	−0.305	0.003	−0.060	0.097	−0.158
GrWt	0.229	0.092	0.179	−0.051	0.589	−0.042
GL	0.017	0.430	0.417	0.042	0.095	−0.295
GB	0.275	0.179	0.370	0.080	−0.129	0.267
KL	−0.120	0.385	0.416	0.200	−0.086	−0.082
KB	0.193	0.117	0.072	0.145	−0.109	0.701
HD	−0.327	−0.314	0.177	0.342	0.298	0.024
MT	−0.330	−0.222	0.096	0.468	0.246	0.123
Till	−0.120	0.029	0.026	−0.352	0.283	0.539
PY	0.390	−0.153	0.165	−0.235	0.345	−0.082

## Pre-breeding lines with pigmented grains and nutritional benefits

**FIGURE 3:**
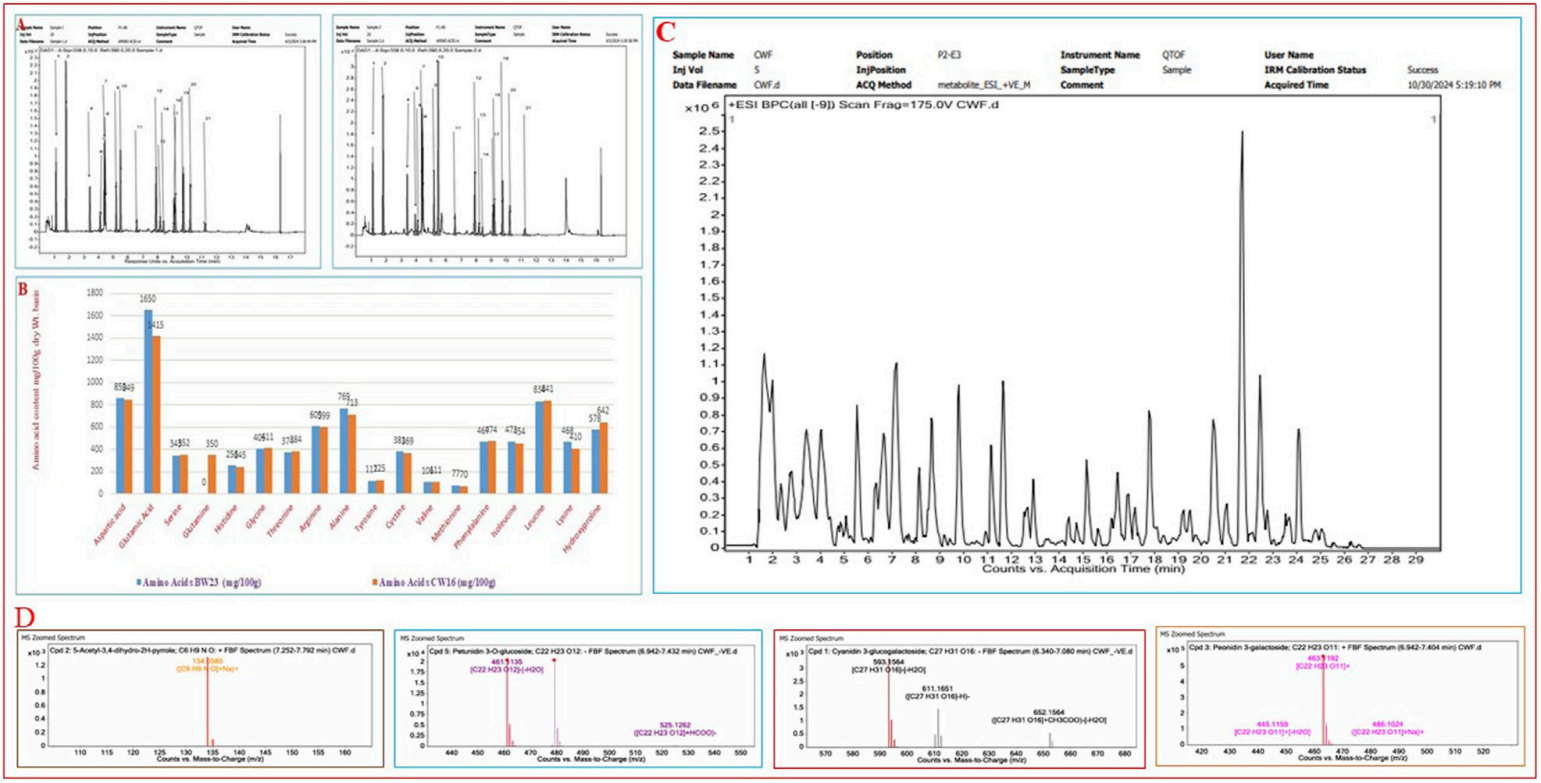
Chromatogram of HR-LCMS-QTOF for the identification of the total free amino acids from two black rice breeding lines (BW23 and CW16) and the qualitative detection of metabolites including anthocyanin pigments (petunidin 3-O-glucoside) from two black rice breeding lines (BW23 and CW16) responsible for black pigmentation and grain quality. **(A)** Chromatogram of amino acids identification. **(B)** Comparative amino acids composition of two lines represented using a bar graph. **(C)** Chromatogram of total metabolites detection from black rice lines (BW23 and CW16). **(D)** MS zoomed spectra of antioxidant and anthocyanin pigments such as 5-acetyl-3,4-dihydro-2H-pyrrole, petunidin 3-O-glucoside, cyanidin 3-O-glucogalactoside, and peonidin 3-galactoside.

Many phenotypic variations were detected in the grain color, which ranged from white, light brown, reddish-brown, brown, deep brown, reddish, red, blackish-red, greenish, blackish-brown, black, to deep black (Figure 1; Supplementary Tables S1 and S2), broadly showing a 9:6:1 ratio. In the present study, we have observed many breeding lines with purple leaf coloration in the CWF cross with black pericarp and black husk color (Figure 1). The purple leaf trait is inherited from the F3 generation, suggesting that the trait has been newly acquired by the breeding lines, although parental lines were devoid of such a trait. The HR-LCMS-QTOF method of metabolomics analysis revealed the detection of several anthocyanin pigment compounds in our black rice breeding lines (BW23 and CW16), such as petunidin 3-O-glucoside, peonidin 3-O-glucoside, peonidin 3-galactoside, cyanidin 3-O-glucogalactoside, and pelargonin, including other 46 metabolite compounds (Figure 3; Table 12). Most common metabolites that were identified were as follows: catechin, oryzanol, gallic acid, caffeic acid, quinic acid, quercetin, 3,5-dihydroxybenzoic acid, rutin, luteolin 4′-O-glucoside, heptadecatrienoic acid, PAB/4-aminobenzoic acid, kaempferol 7-O-glucoside, peganine, maritimetin, mitoxantrone, methyl 2-(10-heptadecenyl)-6-hydroxybenzoate, zinnimidine, azafrin, tubulosine, and other metabolite compounds that have medicinal importance (Table 12). The total amino acid content was quantitatively estimated in the pigmented grain of our pre-breeding lines through the HR-LCMS-QTOF method. The estimation ranged from 8.76 mg/100 g (BW23) to 8.81 mg/100 g (CW16) on dry weight basis, with the following amino acid compositions: aspartic acid, alanine, arginine, cysteine, glutamate, glycine, histidine, isoleucine, leucine, lysine, methionine, phenylalanine, proline (hydroxyproline), serine, threonine, tyrosine, glutamine, and valine (Figure 3; Table 13).

**TABLE 12:** Distinct types (46 components) of metabolites (anthocyanins and others) qualitatively detected from two black rice RIL lines (BW23 and CW16) through HR-LCMS-QTOF and their Nutritional Values depicted.

Compounds name	Formula	Mass	*m*/*z* ratio	Nutritional values and medicinal uses
Catechin	C_15_ H_14_ O_6_	290.0776	289.0702	Antioxidant
Peonidin 3-O-glucoside	C_22_ H_23_ O_11_	463.1197	463.1192	Anthocyanin pigment and antioxidant
Peonidin 3-galactoside	C_22_ H_23_ O_11_	463.1197	463.1192	Anthocyanin pigment and antioxidant
Petunidin 3-O-glucoside	C_22_ H_23_ O_11_	479.1157	478.1083	Anthocyanin pigment and antioxidant
Cyanidin 3-glucogalactoside	C_27_ H_31_ O_16_	611.166	593.1564	Anthocyanin pigment and antioxidant
Pelargonidin	C_15_ H_11_ O_5_	271.0601	271.0631	Anthocyanin pigment and antioxidant
Oryzanol C	C_41_ H_60_ O_4_	616.449	617.4564	Antioxidant
Oryzanol A	C_40_ H_58_ O_4_	602.4503	601.4301	Antioxidant
5-Acetyl-3,4-dihydro-2H-pyrrole	C_6_ H_9_ N O	111.0681	134.058	Arma component, anti-inflammatory, and anticancer effects.
Quinic acid	C_7_ H_12_ O_6_	192.0646	191.0572	Reduce inflammation, improve digestion, and boost the immune system
Quercitrin	C_21_ H_20_ O_11_	448.1022	447.095	Reduce cardiovascular diseases, hypertension, atherosclerosis
Kaempferol 7-O-glucoside	C_21_ H_20_ O_11_	448.1022	447.095	Ovarian, breast, cervical, hepatocellular carcinoma, and leukemia
3,5-dihydroxybenzoic acid	C_7_ H_6_ O_4_	154.0268	153.0196	Antioxidant, anti-aging
Gallic acid	C_7_ H_6_ O_5_	170.0214	169.0143	Antioxidant and anti-inflammatory properties
2,6-Dihydroxyphenylacetate	C_8_ H_8_ O_4_	168.0431	167.0359	Anti-cancerous
alpha-Cotonefuran	C_15_ H_14_ O_6_	290.078	289.0708	Antimicrobial, antihypertensive, anti-ulcer, and anticancer
3-Fluoro-1-(4-hydroxyphenyl)-1-propanone	C_9_ H_9_ F O_2_	168.0579	227.0717	Analgesic
Luteolin 4'-O-glucoside	C_21_ H_20_ O_11_	448.1019	447.0947	Treating hyperuricemia and gouty arthritis
Caffeic acid	C_9_ H_8_ O_4_	180.0417	179.0346	Antioxidant, anti-inflammatory, anticarcinogenic, antibacterial
Genistein	C_15_ H_10_ O_5_	270.0534	315.0516	Antioxidant, anti-inflammatory, anti-obesity, anti-cancer, cardioprotective
Linalyl caprylate	C_18_ H_32_ O_2_	280.2409	279.2337	Treating depression, high blood pressure, and anxiety
Pateamine	C_31_ H_45_ N_3_ O_4_ S	555.3115	554.3052	Anti- cancerous, prevent cell proliferation
(x)-2-Heptanol glucoside	C_13_ H_26_ O_6_	278.1718	323.1708	Bone regeneration, skin care, treating Alzheimer's disease, diabetes
Peganine	C_11_ H_12_ N_2_ O	188.0943	189.1015	Gastro anti-secretory and cyto-protective
Coronaridine	C_21_ H_26_ N_2_ O_2_	338.1985	337.1915	Migraine headaches, hypertension, sexual disorders, or Parkinson's disease
Rutin	C_27_ H_30_ O_16_	610.1509	611.1584	Antifungal and anti-arthritic effects
Maritimetin	C_15_ H_10_ O_6_	286.0469	287.0543	Anti-cancerous
6-C-Galactosylluteolin	C_21_ H_20_ O_11_	448.0991	449.1063	Treat dermatitis
PAB / 4-Aminobenzoic acid	C_7_ H_7_ N O_2_	137.0471	138.0544	Treat infertility in women, rheumatic fever, systemic lupus erythematosus (SLE)
Mitoxantrone	C_22_ H_28_ N_4_ O_6_	444.2032	445.2105	Intravenous anti-cancerous drug
Buprenorphine	C_29_ H_41_ N O_4_	467.2992	468.3067	Treat opoid pain; Pain-killer
Bopindolol	C_23_ H_28_ N_2_ O_3_	380.2105	403.1999	Treatment of cardiovascular diseases
Zinnimidine	C_15_ H_19_ N O_3_	261.137	284.1262	Treat diabetes, obesity and central nervous system disorder
Azafrin	C_27_ H_38_ O_4_	426.276	427.283	Cardio-protective, treatment of ischaemic heart diseases
[7]-Paradol	C_18_ H_28_ O_3_	292.2048	315.1936	Treatment of pancreatic cancer
Tubulosine	C_29_ H_37_ N_3_ O_3_	475.2805	476.2877	Prevent breast cancer
3-Ketosphinganine	C_18_ H_37_ N O_2_	299.2833	322.2726	Anti-bacterial property
Methyl 2-(10-heptadecenyl)-6-hydroxybenzoate	C_25_ H_40_ O_3_	388.2981	411.2874	Fragrance flavouring and soothing agent in oral hygiene products
9Z-Octadecen-12-ynoic acid	C_18_ H_30_ O_2_	278.2238	279.2311	Promoted glucose transport in skeletal muscle
Physalin L	C_28_ H_32_ O_10_	528.2005	529.2079	Anticancer, anti-inflammatory, antiparasitic, antimicrobial, antiviral activities
6-(1,2,3,4-Tetrahydro-6-methoxy-2-naphthyl)-2(1H)-pyridone	C_16_ H_17_ N O_2_	255.1251	256.1325	Anti-inflammatory, analgesic
1-Acetoxy-2-hydroxy-5,12,15-heneicosatrien-4-one	C_23_ H_38_ O_4_	378.2738	401.2632	Antimicrobial
Dexmethylphenidate	C_14_ H_19_ N O_2_	233.1426	256.1319	Used in hyperactive symtoms
Ginsenoyne D	C_17_ H_26_ O_2_	262.1939	285.1831	Stimulates the proliferation of endogenous stem cells
8Z,11Z,14Z-heptadecatrienoic acid	C_17_ H_28_ O_2_	264.2098	287.199	Anti-inflammatory
Lindheimerine	C_22_ H_31_ N O_2_	341.2323	364.2217	Hypoglycaemic activity

**TABLE 13:** Total amino acids in black rice breeding lines (mg/100 g dry weight basis) quantitatively detected by using the HR-LCMS-QTOF method.

Amino acids	Aromatic black rice breeding lines (black pericarp)	
	BW23	CW16
	(mg/100 g)	(mg/100 g)
Aspartic acid	859	849
Glutamic acid	1,650	1,415
Serine	343	352
Glutamine	0	350
Histidine	256	245
Glycine	405	411
Threonine	374	384
Arginine	609	599
Alanine	769	713
Tyrosine	117	125
Cystine	381	369
Valine	106	111
Methionine	77	70
Phenylalanine	467	474
Isoleucine	473	454
Leucine	834	841
Lysine	468	410
Hydroxyproline	578	642

## Discussion

### Yield enhancement with pigmented grain quality in both the RIL populations

In the present study, two distinct RIL populations comprising 100 genotypes (BWF and CWF) were developed and characterized using 15 yield-related agro-morphological traits that indicated the presence of gigantic variability for these traits. ANOVA was employed to assess the significance of phenotypic variation in 15 yield-related agronomic traits across the BWF and CWF populations. The results indicated significant genotypic differences (p < 0.001) for most traits, confirming the presence of substantial genetic variation introgressed from *O. rufipogon* (significant at p < 0.001) (Table 1). The results were consistent with the general notion that the larger the divergence between the parental genotypes is, the higher the heterosis in crosses will be (Falconer, 1964) (Tables 2 and 3). The PY was recorded with the mean values 14.95 g, 22.65 g, and 10.66 g in the control black Chakhao, parent 1 Badshabhog, and parent 2 wild rice (*O. rufipogon*), respectively, whereas BWF RIL genotypes displayed PY that ranged from 5.00 g to 61.89 g with the mean value of 28.62 g (Table 2). In the case of CWF RIL genotypes, PY was recorded with mean values 14.95 g, 24.79 g, and 10.66 g in the control black Chakhao, parent 1 Chenga, and parent 2 wild rice (*O. rufipogon*), respectively, and the CWF RIL genotypes displayed PY that ranged from 10.66 g to 43.87 g with a mean value of 22.80 g (Table 3). At least 15 breeding lines out of the 100 BWF RIL populations were considered as promising lines due to better performance related to PY during two kharif seasons (2022 and 2023) with early maturity times (125 days–135 days) than the control black variety Chakhao, Manipur (153 days and plant height of 156.55 cm). The following breeding lines showed high PY: BW6 (33.48 g), BW18 (40.37 g), BW23 (61.89 g), BW24 (56.59 g), BW25 (51.93 g), BW26 (34.19 g), BW33 (32.48 g), BW44 (31.70 g), BW50 (42.74 g), BW77 (37.67 g), BW83 (50.22 g), BW88 (35.94 g), BW90 (43.93 g), BW91 (26.30 g), and BW99 (60.89 g) from the BWF population and the genotypes CW1 (37.99 g), CW11 (37.11 g), CW16 (43.87 g), CW20 (25.70 g), CW23 (26.32 g), CW39 (32.59 g), CW69 (27.86 g), CW78 (32.44 g), CW79 (37.76 g), CW80 (29.11 g), CW90 (33.65 g), CW94 (29.62 g), CW95 (22.78 g), CW97 (25.50 g), and CW98 (23.44 g) from the CWF population (Tables 2 and 3). Our finding was consistent with the earlier report that yield was enhanced when local rice Chakhao Poireiton (purple) was crossed with HYV Sahbhagi Dhan (white) and Shasharang (light brown) (Lap et al., 2024).

### Genetic variability parameters

A wide range of phenotypic diversity was observed in both the RIL populations and showed transgressive segregation with respect to GL, grain number per panicle, GrWt, PnL, and PnWt (Figure 1; Tables 2 and 3; Supplementary Tables S1 and S2). The results signify that both the RIL populations (BWF and CWF) have shown substantial genetic variation among the genotypes (Tables 2 and 3). The nature and magnitude of genetic divergence prevailing in both the RIL populations (BWF and CWF) were estimated by various multivariate statistical tools, such as genetic variability parameters (broad-sense heritability), Mahalanobis D^2^ statistic, PCA, and cluster analysis using standard formulae. The present results were consistent with the earlier findings that genetic divergence prevailed in our pre-breeding RIL populations (Faysal et al., 2022; At-Ul-Karim et al., 2022; Mondal et al., 2024; Mudhale et al., 2024; Bordoloi et al., 2024). Success in crop improvement generally depends on the magnitude of genetic variability and/or diversity and the extent to which the desirable characteristics are heritable (Mondal et al., 2024). Heritability is crucial in determining a trait’s response to selection and predicting the transmission of desirable characteristics from parents to offspring during breeding (Acquaah, 2009). The traits with high heritability such as PH, active tillering, filled grain per plant, GL, FLW, PnL, and GrWt have been widely reported to effect rice yield (At-Ul-Karim et al., 2022). The yield of rice is controlled by three key components: the number of effective panicles, the number of grains per panicle, and grain weight (Zheng et al., 2024). Agronomic traits such as GrWt and PH have been widely used for the improvement of rice yield in breeding programs (Hernandez-Soto et al., 2021). In this study, moderate-to-high heritability (74.42%–98.71%) was observed in both the RILs F_8:9_ (BWF and CWF), indicating moderate-to-high level of genetic control of the traits associated with yield parameters (At-Ul-Karim et al., 2022; Hernandez-Soto et al., 2021; Zheng et al., 2024), and it subsequently showed increased yield in the RILs during field trials in two kharif seasons (2022 and 2023) (Tables 2–4). RIL genotypes (BWF and CWF) showed high yield potential due to high heritability of all the yield-enhancing characters evaluated in the present study (PH, active tillering, filled grain per plant, GL, FLW, PnL, and GrWt) (Tables 2 and 3). High heritability (>80%) with high GA (>20) was observed for the traits PH, PnL, GrPn, GrWt, and PY in BWF lines and PH, PnL, GrPn, and KB in CWF lines, suggesting additive gene action for the characteristics and that these traits could contribute largely to the yield improvement of pre-breeding lines (Tables 2–4). High heritability (H %) for yield-related traits in the BWF population (H % = 80 for PH, GrWt, PY, GrPn, and PnL) indicated strong genetic control, which is likely due to novel alleles from *O. rufipogon*. Similarly, the CWF population exhibited high heritability for the traits PH, PnL, GrPn, and KB (H % = 80), suggesting that introgression enhanced the genetic contribution to phenotypic variation, facilitating selection for improved traits. High heritability (>90%) in combination with the high score of genetic advance as percent of mean (>40%) was also observed for the traits PnWt, GrPn, and PY in the CWF RIL population, indicating strong additive genetic effects for increasing yield (Table 4). High heritability indicates strong genetic control, and genetic advance highlights the potential for trait improvement, which are all driven by *O. rufipogon* alleles. Many genes/QTLs of yield-enhancing traits (spikelet number, grain number per panicle, PnL, GrWt, grain size, grain yield, and GL) were identified and introgressed into the elite rice germplasm from the progenitor wild rice for enhancing the yield characters (McCouch et al., 2007; Gaikwad et al., 2021; Roy and Shil, 2020a; Xu et al., 2021; Malik et al., 2022; Seck et al., 2023). Significant genetic gain can be achieved in improving varieties by utilizing novel genes of the neglected wild rice to restore the genetic diversity and allelic variation lost during domestication (Siddiq and Vemireddy, 2021; Eizenga et al., 2024). Superior genes/QTLs of agronomic importance from wild rice (*O. rufipogon*) can be directly incorporated into breeding programs to generate pre-breeding material, which will serve as a valuable germplasm resource for rice breeding (Henry, 2022; Zhang J. et al., 2022; Bedford et al., 2023; Zheng et al., 2024). Similarly, these types of yield-enhancing traits (allelic variants/genes/QTLs) must have been introgressed into our pre-breeding lines from untapped wild rice (*O. rufipogon*); otherwise, both the RILs (BWF and CWF) would not show such high heritability with high genetic advance for yield characteristics, and they would have also exhibited high heterotic phenotypic features (Tables 2–4). Increased phenotypic variance observed in both the RIL populations compared with the parental lines could be attributed to the increased level of transgression of these yield-related components and gene interactions (Tables 2 and 3). Therefore, our pre-breeding materials (RILs) are valuable resources for rice improvement programs that may provide a powerful tool for broadening the genetic base of breeding materials to improve rice productivity, including climate change-resilient phenotypes along with high yield and grain quality. The improvement of rice grain quality has become an important breeding target in almost all rice breeding programs since the early 1980s (Mackill and Khush, 2018). Some of the grain qualities (ASV, GT, GC, and aroma) were evaluated in the present study and are depicted in Supplementary Tables S1 and S2.

### Clustering and dendrogram construction

The RIL population of BWF was grouped into 5 clusters according to the mean cluster values based on 15 traits, and the CWF RIL population was grouped into four clusters, indicating the relationship among the 100 genotypes (Table 5; Figures 2H, G). Our present findings are consistent with the earlier studies of Ahmad et al. (2015) and Bordoloi et al. (2024).

### Mahalanobis D^2^ test for genetic diversity assessment in BWF and CWF

The Mahalanobis D^2^ test is a multivariate statistical tool used to measure the genetic divergence or distance between genotypes based on multiple traits. It is particularly valuable for assessing diversity based on Mahalanobis D^2^ values. Genotypes with large D^2^ values are genetically divergent. Mahalanobis D^2^ distance values can be used to group genotypes into several clusters using Tocher’s method. The BWF RIL population was grouped into 7 clusters and the CWF RIL population was grouped into eleven distinct clusters based on Tocher’s method using Mahalanobis D^2^ distance values, reflecting the successful introgression of wild alleles (Tables 6 and 7). The average intercluster distances were observed to be greater than the average intra-cluster distances, suggesting that the genotypes of both the RIL populations (BWF and CWF) possess a greater degree of genetic diversity. The largest intercluster distances were found between clusters IV and VII (25,865.50) in BWF lines, indicating that genotypes in cluster IV were far diverse from those of cluster VII. The largest intercluster distances (25,817.49) were found between clusters II and IV in the CWF RIL population. The maximum intercluster distance indicated wide diversity, whereas the minimum suggested a close relationship between the groups (At-Ul-Karim et al., 2022). Genotypes with the largest genetic distance in yield-attributing parameters would result in the complementation of gene effects in the hybridization program, and these were detected in the present RILs (Tables 6 and 7). Out of the 15 agro-morphological traits studied, only 5 traits (GrPn, GrWt, KB, GB, and PH) provided the maximum contribution (65.50%) toward the total divergence in the BWF RIL population (Table 7). The contribution toward the total variation was the maximum for GrPn (19.00), followed by GrWt (13.90), KB (13.60), GB (10.50), GL (8.80), PH (8.50), PnWt (5.30), KL (5.00), and PY (4.30) in the CWF RIL population (Table 8). Overall, we have observed that the main characteristics that helped express our study’s diversity were the traits PH, GrPn, GrWt, GB, KL, PY, and KB (Tables 8 and 9). These traits should be taken into consideration while selecting parents for hybridization. Mahalanobis distance-based clustering pattern of both the RIL genotypes (BWF and CWF) into several groups confirmed the quantum of diversity present in the developed pre-breeding lines and provides a scope for its exploitation through breeding for yield improvement in rice (Tables 6–9). We know that the transfer of genes governing desirable traits from wild relatives to cultivated rice is an important strategy in rice breeding (Sanchez et al., 2013; Qiao et al., 2016). The narrow genetic base of modern rice varieties has led to yield plateaus, making it essential to introduce genetic diversity to overcome these barriers. Moreover, the genetic bottleneck that occurred during the domestication of cultivated rice from its immediate ancestral progenitor of wild rice *O. rufipogon* has played a major role in reducing allelic diversity by at least 50%–60% in cultivated rice than in wild rice *O. rufipogon*, leading to the loss of genetic variability with yield potentiality (Xiao et al., 1996; Tanksley and McCouch, 1997; Brar and Khush, 2006). Wild rice *O. rufipogon* has been considered as a reservoir of many untapped gene/QTLs for important agronomic traits, such as yield, quality, nutritional characteristics, and resistance to biotic and abiotic stresses, and it can be utilized in the pre-breeding program for broadening the genetic base of the released varieties to break the yield plateaus (Tanksley and McCouch, 1997; McCouch et al., 2007; Tester and Langridge, 2010; Sanchez et al., 2013; Brar and Khush, 2018). The transfer of genes controlling desirable traits (yield and grain quality) from the wild relatives *O. rufipogon* to cultivated rice is an important strategy in rice breeding (Tian et al., 2006; Huang et al., 2012B; Qiao et al., 2016; Gaikwad et al., 2021; Zhang B. et al., 2022). Pre-breeding facilitates the introgression of desirable traits such as yield potential, nutritional quality, and resistance to biotic and abiotic stresses (Rao et al., 2020; Bin Rahman and Zhang, 2022; Alam et al., 2024). In this study, we corroborate the results of earlier findings that wild rice serves as a valuable genetic resource for enhancing rice varieties via widening the genetic base through introgression of hidden alien gene/QTLs for high yield potential (Tanksley and McCouch, 1997; Henry, 2022; Bedford et al., 2023; Zheng et al., 2024). A complex trait such as grain yield is controlled by many genes along with being influenced by the environment and is related to other traits such as plant types, growth duration, and other yield-component traits (Mudhale et al., 2024; Bordoloi et al., 2024). The present reports are consistent with the earlier analyses that higher intercluster distances existed in the breeding lines, indicating wide trait variability and genetic divergence (Faysal et al., 2022; At-Ul-Karim et al., 2022; Mondal et al., 2024). Both the RIL populations (BWF and CWF) have shown wide genetic divergence in respect to the 15 characters studied, signifying that the genetic base has broadened as a result of interspecific hybridization through the introgression of untapped genetic components from the underutilized wild rice (*O. rufipogon*) germplasm of India (Tables 2 and 3). The yield-enhancing associated traits such as spikelet number, grain number, grain size, grain weight, and PnL have been introgressed into the populations developed from crosses of *O. sativa* × *O. rufipogon*, making *O. rufipogon* an ideal germplasm for mining yield-enhancing loci (McCouch et al., 2007; Malik et al., 2022; Gaikwad et al., 2021). These traits also contribute toward increasing the creation of high genetic variation and diversity in our pre-breeding RILs (BWF and CWF) (Tables 6–9).

### Principal component analysis

PCA was used to explore the genetic diversity and population structure in the BWF and CWF populations. It was utilized to categorize all the yield-attributing traits into distinct PCs, thereby revealing the individual traits’ contributions to genetic divergence. The first four components in our study were considered the primary PCs as they showed the greatest variability with eigenvalues greater than >1 in the BWF RIL population (Table 10) and six components in the CWF population (Table 11). It showed that the maximum variation was present in the first two PCs (BWF and CWF), and hence, selection of genotypes from these PCs will be useful for obtaining higher genetic variation with higher yields (Figure 2; Tables 10 and 11). Breeders can use selection to influence such significant traits in the divergence analysis of BWF and CWF RIL populations. In this study, only PH and PnWt exhibited positive values in the first four PCs associated with divergence in the BWF RIL population (Table 10). PC1 and PC2 in the PCA-bi-plot diagram showed the dispersion and nature of diversity for both variables and genotypes (Figure 2). The cumulative variance of 73.74% by the first four axes with an eigenvalue > 1.00 indicates that the identified traits significantly influenced the RIL’s phenotype and could effectively be used for selecting among them in the BWF genotypes (Table 10). The bi-plot analysis showed the relationships between the morphological traits among the tested RIL genotypes of BWF and CWF (Figure 2). The traits influencing PC1 were PnWt, GrWt, GL, GB, KL, and PY. These results also support the GCV estimates for PnWt, GrWt, GL, GB, KL, and PY; the first three traits, along with GrPn, KB, and PH, also corroborated Mahalanobis distance-based divergence in the present study. PCA and Mahalanobis D^2^ analyses further validate the introduction of novel genetic diversity, as RILs exhibit distinct genetic profiles and increased divergence from the cultivated parents. These findings demonstrate that *O. rufipogon* introgression successfully broadens the genetic base, enhancing the diversity and the potential for developing resilient, high-yielding rice varieties (Tables 2 and 3). Based on the comparison of the 100 RILs of BWF based on PCA bi-plot analysis, the RILs BW18, BW23, BW24, BW25, BW44, BW52, BW77, BW83, BW88, BW90, and BW99 were superior for PnWt, GrWt, PY, GL, GB, and KL. Hence, these results of PCA will be of great benefit to the breeder for identifying parents and the selection of characters for future hybridization programs for varietal improvement. The genetic diversity of the breeding lines (BWF and CWF) was clarified, and component traits contributing to variability were broken down through the combination of PCA; this could provide the framework for a well-run hybridization program. The length of a trait’s vector in PCA represents its contribution to the overall divergence; the longer the vector, the larger the contribution (Figure 2). All the traits exhibited the maximum length and contributed maximally to the total diversity. These results were in conformity with the findings of Bhuvaneswari et al. (2020) and Mondal et al. (2024). The dispersion of RILs across PCA axes confirmed the introduction of novel genetic diversity, broadening the genetic base beyond that of *O. sativa*.

## Pre-breeding lines with pigmented grains and nutritional benefits

Wild species *O. rufipogon* is extensively used for the mining of new genes for biotic/abiotic stresses and high-value QTLs for yield and grain quality traits (Gaikwad et al., 2021; Malik et al., 2022). Pre-breeding (*O. sativa* × *O. rufipogon*) has been utilized not only for improving the qualitative and quantitative traits but also for introgressing new useful variability, which recognizes its potential as a valuable reservoir of genetic variation (Tanksley and McCouch, 1997; Brar and Khush, 2018; Roy and Shil, 2020b; Gaikwad et al., 2021; Henry, 2022; Malik et al., 2022). In the present investigation, the most innovative and novel genetic change that we observed in the progeny populations (BWF and CWF) was the appearance of rice lines containing black pericarp. We also observed many breeding lines with purple leaf coloration in the CWF cross with black pericarp and black husk color (Figure 1; Supplementary Tables S1 and S2). The purple leaf trait is inherited from the F3 generation, suggesting that the trait has been newly acquired by the breeding lines, although parental lines were devoid of such a trait.

This unique finding is consistent with the earlier results that new useful genetic variation may be created in the progeny population when crossed with wild progenitor *O. rufipogon* (Tanksley and McCouch, 1997; Sanchez et al., 2013; Brar and Khush, 2018; Gaikwad et al., 2021; Xia et al., 2021; Malik et al., 2022). Thus, the most innovative and novel genetic change that we observed in the progeny populations (BWF and CWF) was the appearance of rice lines containing black pericarp; however, the parental lines were non-black (*O. rufipogon*, red grain; Chenga, brown; and Badshabhog, white) with only the green leaf character. Many phenotypic variations were detected in the grain color (pericarp pigmentation), which ranged from white, light brown, reddish-brown, brown, deep brown, reddish, red, blackish-red, greenish, blackish brown, black, to deep black (Figure 1; Supplementary Tables S1 and S2), broadly showing a 9:6:1 ratio (polymeric gene interaction) in the progeny populations. Other types of segregation ratios such as 9:7 (complementary gene interaction) and 9:3:4 (supplementary gene interaction) were also reported earlier (Devi et al., 2020; Lap et al., 2024). The exceptional range of color variations also supports the view that grain color is of polygenic inheritance in nature and controlled by many genes or quantitative trait loci (QTL) or/involves as yet unidentified genes (Oikawa et al., 2015; Ham et al., 2015; Devi et al., 2020; Pham et al., 2024). Our present study was concomitant with the earlier analysis that domestication and population divergence in crops produce considerable phenotypic changes, reflecting their genomic evolutionary trajectories, particularly in structural variants (SVs) and gene expression (Shang et al., 2022; Xu et al., 2024b). Following this notion, a known genetic construction (Kala4 gene with LINE1 insertional mutation) that reappears through unfolding the hidden genetic components or new genetic constructs acquired *de novo* by exchanging genomic segments during meiotic recombination/chromosomal rearrangement of SVs leads to acquired neofunctionalization to form black pericarp. The nutritional quality of rice is determined by the levels of starch, protein, lipids, minerals, vitamins, and phytochemicals (Senguttuvel et al., 2023). Pigmented rice varieties (brown, red, and black) are gaining popularity among consumers due to their nutritional health benefits (Shao et al., 2018; Mendoza-Sarmiento et al., 2023; Xie et al., 2024; Zhu et al., 2024; Idrishi et al., 2024; Sakulsingharoj et al., 2024; Gogoi et al., 2024), and market demands are expected to increase (Kushwaha, 2016; Ito and Lacerda, 2019; Bhuvaneswari et al., 2023). Pigmented rice accumulates various types of secondary metabolites, such as phytosterols, polyphenols, flavonoids, anthocyanins, proanthocyanidins, vitamins, and micronutrients (Shao et al., 2018; Mendoza-Sarmiento et al., 2023; Zhu et al., 2024; Idrishi et al., 2024), which are recognized to have a high nutritional value and medicinal properties, with antioxidant, antimutagenic, anticancer, antiviral, antidiabetic, anti-inflammatory, and antiaging potentialities (Mbanjo et al., 2020; Das et al., 2023; Sakulsingharoj et al., 2024; Gogoi et al., 2024). However, pigmented rice landraces often have lower yields and less favorable agronomic traits, necessitating pre-breeding to integrate their nutritional benefits into high-yielding, resilient varieties. The nutritive value of pigmented rice is greatly influenced by genetics, genotypic variation, and environmental factors (Sweeney et al., 2006; 2007; Furukawa et al., 2007; Gross and Zhao, 2014; Tiozon et al., 2023; Zhu et al., 2024), along with several external influences such as soil fertility status, the degree of milling, and the method of preparation before consumption (Gogoi et al., 2024). Through HR-LCMS-QTOF metabolomics profiling and amino acid identification from the RIL genotypes (BW23 and CW16), we confirm that our black rice lines are rich in phytonutrients (polyphenols, flavonoids, anthocyanins, oryzanol, catechin, quercitrin, kaempferol 7-O-glucoside, 5-Acetyl-3,4-dihydro-2H-pyrrole, and others), which have health benefits (Figure 3; Table 12). A total of 46 most vital metabolites were identified through the HR-LCMS-QTOF method and the total amino acids profiling carried out on black rice lines (BW23 and CW16) (Tables 12 and 13). Several anthocyanin pigment compounds were qualitatively identified from our black rice lines, such as petunidin 3 O-glucoside, peonidin 3-O glucoside, peonidin 3-galactoside, cyanidin 3-O-glucogalactoside, and pelargonin, confirming that the black color is due to the presence of anthocyanin pigments (Figure 3; Table 12). The most common metabolites that were identified were as follows: catechin, oryzanol, gallic acid, caffeic acid, quinic acid, quercetin, 3,5-dihydroxybenzoic acid, rutin, luteolin 4′-O-glucoside, heptadecatrienoic acid, PAB/4-aminobenzoic acid, kaempferol 7-O-glucoside, peganine, maritimetin, mitoxantrone, methyl 2-(10-heptadecenyl)-6-hydroxybenzoate, zinnimidine, azafrin, tubulosine, and other metabolite compounds that have nutritional value and medicinal importance (Table 12). Our present results are consistent with the previous report about the health benefits and medicinal uses, with antioxidant, antimutagenic, anticancer, antiviral, antidiabetic, anti-inflammatory, and antiaging potentialities (Mbanjo et al., 2020; Das et al., 2023; Sakulsingharoj et al., 2024; Gogoi et al., 2024). The total amino acid estimation ranged from 8.76 mg/100 g (BW23) to 8.81 mg/100 g (CW16) based on the dry weight, with the following amino acid compositions: aspartic acid, alanine, arginine, cysteine, glutamate, glycine, histidine, isoleucine, leucine, lysine, methionine, phenylalanine, proline (hydroxyproline), serine, threonine, tyrosine, glutamine, and valine (Figure 3; Table 12). The amino acid profile of our rice shows that it is high in glutamic and aspartic acid, whereas lysine is the limiting amino acid, which is similar to that in another analysis (Carcea, 2021). In the present study, glutamic acid was found in the highest amount (1,650 mg/100g) in BW23, and methionine was found in the lowest amount (70 mg/100g) in CW16. The taste is better in CW16 due to the presence of glutamine (358 mg/100 g). Proline (as hydroxyproline) was quite high in the CW16 (642 mg/100 g) and in BW23 lines (578 mg/100 g), leading to a popcorn-like aroma. Proteins containing amino acids such as lysine, leucine, isoleucine, and threonine are considered high-quality proteins (Min et al., 2019; Liyanaarachchi et al., 2020; Tyagi et al., 2022; Jayaprakash et al., 2022). Our results showed that both the black rice breeding lines (BW23 and CW16) are nutritionally enriched due to the presence of high-quality proteins in the endosperm containing amino acids such as lysine, leucine, isoleucine, and threonine (Table 13). Our present investigation is consistent with earlier reports that the newly developed black rice breeding lines are nutritionally enriched in respect to the high-quality amino acids content (Min et al., 2019; Liyanaarachchi et al., 2020; Tyagi et al., 2022; Jayaprakash et al., 2022) and other important nutraceuticals, that is, oryzanol, anthocyanin, catechin, quercitrin, kaempferol 7-O-glucoside, and 5-acetyl-3,4-dihydro-2H-pyrrole (Tables 12 and 13) (Ahmad et al., 2015). A similar pattern of metabolites was reported by previous studies in pigmented rice varieties (Bhuvaneswari et al., 2020; Zhu et al., 2024).

## Conclusion

Pre-breeding is indispensable for rice improvement programs, addressing critical challenges such as climate change, food security, malnutrition, and sustainability. By unlocking the genetic potential of wild relatives (*O. rufipogon*), we have successfully developed two wide-ranging pre-breeding populations (F_8:9_) containing 100 RILs (each BWF and CWF) in the genetic background of local indica cultivars Badshabhog and Chenga. The analyses of ANOVA, broad-sense heritability (H%), genetic advance, PCA, and Mahalanobis D^2^ test statistics collectively demonstrate that the genetic base of the BWF and CWF RIL populations has been significantly broadened through the alien introgression of untapped hidden genes from underutilized wild rice (*O. rufipogon*). These findings support the use of *O. rufipogon* as a valuable resource for rice improvement, offering novel alleles to overcome the limitations of a narrow genetic base in cultivated rice. At least 15 breeding lines from each RIL populations (BWF and CWF) were regarded as promising lines due to high-yield performance during two kharif seasons (2022 and 2023), with early maturity times (125 days–135 days) as compared to control black variety Chakhao, Manipur (153 days and plant height of 156.55 cm). The PY is 14.95 g in the control Chakhao, whereas our black rice lines showed an average 30 g PY with short PH (130 cm–145 cm). Novel and unique traits, such as black pericarp and purple leaf, were discovered in the progeny lines, which were inherited persistently in the RIL populations (F_8:9_), confirming the earlier concept that domestication and population divergence in crops produce considerable phenotypic changes, reflecting their genomic evolutionary trajectories, particularly in structural variants (SVs) and gene expression. Anthocyanin pigments responsible for black pericarp color such as petunidin 3-O-glucoside, peonidin 3-O-glucoside, peonidin 3-galactoside, cyanidin 3-O-glucogalactoside, and pelargonin were qualitatively identified from the black rice lines (BW23 and CW16) through HR-LCMS-QTOF, signifying that genetic components related to the anthocyanin biosynthetic pathway(s) were activated in the pre-breeding materials; otherwise, the black colored pericarp would not be possible. This study supports the earlier concept that pre-breeding (*O. sativa* × *O. rufipogon*) has the potential to be a valuable source of genetic variation because it not only improves qualitative and quantitative traits with a great deal of genetic diversity but also introduces new functional variability. Metabolomics analysis (HR-LCMS-QTOF) identified 46 different phytonutrients metabolites in black rice lines (BW23 and CW16), which indicates the grain quality improvement with medicinal values and health benefits. Our pre-breeding lines can be used as an important genetic resource for improving black rice varieties for food and nutritional security.

## Data Availability

The original contributions presented in the study are included in the article/supplementary material, further inquiries can be directed to the corresponding author.

## Abbreviations

Awn, awn length; BWF, Badshabhog × wild rice progeny; CWF, Chenga × wild rice progeny; DUS, guideline-distinctiveness uniformity and stability; FLL, flag leaf length; FLW, flag leaf width; GrPn, grain per panicle; GL, grain length; GB, grain breadth; GrWt, 1000 grain weight; HD, heading date; PC, pericarp color; PH, plant height; PY, single plant yield; PnL, panicle length; Till, active tiller number; HYV, high-yielding variety; Kala, key activator loci for anthocyanin; LINE1, long interspersed nuclear element-1; MT, maturity time in days; QTL, quantitative trait locus; PCA, principal component analysis; PCV, phenotypic coefficient of variation; GCV, genotypic coefficient of variation; HR-LCMS-QTOF, high-resolution-liquid chromatography–mass spectrometry-QTOF.
